# Evaluation of a co-produced delirium awareness programme for undergraduate nursing students in Northern Ireland: a pre-test/post-test study

**DOI:** 10.1186/s12912-020-00427-9

**Published:** 2020-04-26

**Authors:** Gary Mitchell, Clare McVeigh, Susan Carlisle, Christine Brown-Wilson

**Affiliations:** Queen’s University Belfast, School of Nursing and Midwifery, Medical Biology Centre, 97 Lisburn Road, County Antrim, Belfast, Northern Ireland BT9 7BL

**Keywords:** Delirium, Nurse education, Nursing student, Nursing, Co-design, Co-production

## Abstract

**Background:**

Delirium is recognised internationally as a common disorder that causes acute deterioration in a person’s cognitive abilities. Healthcare professionals play a key role in the early identification and management of delirium and effective education can support timely recognition and treatment. There is currently a lack of research exploring the delirium education provided to undergraduate nursing students. The aim of this study was to evaluate the effectiveness of a co-produced delirium awareness programme on undergraduate nursing students in Northern Ireland.

**Methods:**

The intervention was a 2-h delirium workshop, delivered in April 2019, to a convenience sample of year one undergraduate nursing students (*n* = 206) completing a BSc Honours Nursing degree programme in a Northern Ireland University. The workshop focused on four core elements: defining delirium, reflecting on practice, recognition of delirium and management of delirium. Participants completed a 35-item true-false Delirium Knowledge Questionnaire (DKQ) at baseline and post intervention using Socrative, a cloud-based student response system. In addition, students also completed a short questionnaire at baseline and post-workshop, designed by the authors, to ascertain perceived confidence about caring for people with delirium. Data were analysed using paired t-tests and descriptive statistics.

**Results:**

In the DKQ, Scores were normally distributed around the mean at baseline (71.89%) and post intervention (81.89%). Students improved across all three core areas in the post-test questionnaire, demonstrating improvements in knowledge about symptoms of delirium (7.32% increase), causes and risk factors of delirium (17.91% increase) and management of delirium (5.72% increase). In relation to perceived confidence, students reported a 60.20% increase in confidence related to recognition of delirium, a 49.51% increase in relation to delirium management and a 45.04% increase their ability to communicate about delirium. Both questionnaires were statistically significant (*P* < 0.01).

**Conclusions:**

A 2-h workshop on delirium improved first year student nurse knowledge about delirium. Nursing students expressed that this approach to delirium education enabled collective thinking about how knowledge could be transferred into individual practises. Students also stated that learning incorporating the voice of the person who has experienced delirium, was an effective and powerful way to deliver education.

## Background

Delirium is a common disorder that is characterised by a rapid deterioration, hours or days, of cognitive function triggered by a medical disorder or environmental change [1]. Delirium can be distressing affecting a person’s attention, thinking, orientation, memory, emotions, sleep and behaviour, potentially leading to long-lasting physical and cognitive impairment or premature death [2]. It is estimated that delirium occurs in approximately 30% of hospitalised patients, up to 80% of mechanically ventilated patients and up to 60% of residents living in a care home setting [3]. Delirium most commonly occurs in young children and older people [2, 3]. However, despite delirium becoming a growing global healthcare concern [4–6], it is frequently under diagnosed in primary, secondary and tertiary care [7, 8]. Importantly, delirium is a symptom of acute illness that is often avoidable and reversible [9, 10]. Early recognition of delirium is essential to enable healthcare professionals to identify reversible causes and appropriate intervention tailored to the individual needs of the person.

Healthcare professionals often lack the necessary confidence, knowledge and skills to optimally prevent, identify and manage delirium [11]. Non-detection of delirium is a consequence of insufficient knowledge, poor utilisation of available screening or assessment tools and the diverse ways in which delirium can present in patients [12]. Internationally, these challenges have been recognised and common themes relate to knowledge deficits about: hyperactive and hypoactive delirium, difficulty in differentiating between delirium and dementia, poor recognition of the fluctuating nature of delirium and inability to identify appropriate methods of support to patients and family members effected by delirium [13, 14].

Research suggests that delirium education, targeting front-line health care professionals, has the potential prevent delirium and to reduce the impact of the condition on patients [12]. Delirium education can effectively support healthcare professionals, including nurses, to significantly improve their knowledge of this condition, thereby leading to the timely recognition and treatment of delirium [15–17]. While there have been a number of high-quality research studies demonstrating improved outcomes post delirium education [18, 19], there are limited studies that examine the effects of delirium education on nursing students [20]. Therefore this study aimed to determine the effectiveness of a face-to-face delirium educational package in increasing nursing students’ knowledge of delirium.

## Methods

### Design/setting/population

A pre-test/post-test study was conducted in a convenience sample of first year undergraduate nursing students (*n* = 206) at a university in Northern Ireland. All undergraduate nursing students (*n* = 313) in the first year of the University’s BSc Hons Degree Nursing programme were eligible for inclusion in this study. At the time of delirium-education all participating students had undertaken two clinical placements, each lasting 6 weeks, as part of their nursing programme. All participants were enrolled as a nursing student on one of the four programmes; adult nursing, mental health nursing, children’s nursing or learning disability nursing. All students received the same version of the intervention.

### Intervention

In 2018, the authors worked in partnership with specialist nurses at Royal College of Nursing (United Kingdom) and service users with lived experiences of delirium to develop a two-hour face-to-face learning package for first-year student nurses about delirium. This collaborative co-production approach has been growing over the past number of years in inter-professional education [21]. Collaborative co-production is a way of ensuring that the voice, and lived experience, of the service user is part of healthcare professional education [22]. Co-production challenges the assumption that service users are passive in their care and can actively contribute to curriculum development [23]. Co-production also serves to enrich student learning as it seeks to illustrate the human perspective of illness, for example how it feels to experience delirium [24]. The development of the intervention was an iterative process involving face-to-face meetings, skype interviews and regular use of online discussion forums over 6 months. These meetings brought together expertise and insight from three main areas:

1. Lived Experience of Delirium: from people who had experienced delirium
2. Holistic Delirium Care: from specialist nurses with clinical expertise in delirium
3. Nursing Education: from nurse academics experienced in developing nurse education

The intervention was a 2-h workshop on delirium which was delivered by nurse academics in April 2019 to groups of 15–20 students at a time. These workshops all adopted the same format and can be viewed in supplementary file 1. The intervention was comprised of a face to face workshop that incorporated didactic teaching, case scenarios, reflective practice and media clips [25–27]. The delirium workshop focused on four core elements: defining delirium, reflecting on current practices, recognition of delirium and management of delirium. A copy of the 2-h workshop can be viewed in supplementary file 1. Table 1 provides an overview of the themes presented during the delirium workshop.

**Table 1:** Delirium Workshop Overview

Core Section	Themes
1. What is Delirium?	▪ Background to Delirium
	▪ Prevalence of Delirium
	▪ Symptoms of Delirium
	▪ Types of Delirium
	▪ Delirium vs. Dementia
	▪ Causes of Delirium
	▪ Environmental Factors
2. Reflection on Practice	▪ Video-Based Case Scenario on Hypoactive Delirium
	▪ Video-Based Case Scenario on Hyperactive Delirium
3. Recognition of Delirium	▪ NICE Recommendations on Assessment and Diagnosis of Delirium.
	▪ The 4 A’s Test
4. Management of Delirium	▪ The Lived Experience of Delirium
	▪ Nursing Management of Delirium
	▪ Prevention of Delirium
5. Post Workshop Reflective Exercise	▪ Post Workshop Reflective Exercise Summarising Key Learning Points.

### Data collection

A 35-item true-false Delirium Knowledge Questionnaire (DKQ), developed by Detroyer [28] and colleagues, was administered to students immediately before and immediately after the workshop. This 35-item questionnaire was based on a previous questionnaire by Hare and colleagues [29]. The DKQ was designed to assess healthcare professional knowledge about delirium and is divided into three main categories: 1) knowledge related to the presentation, symptoms and outcomes of delirium (*n* = 10 items), 2) knowledge related to the causes and risk factors of delirium (*n* = 11 items), and 3) knowledge about delirium prevention and management strategies (*n* = 14 items). The total DKQ score is the sum of the correct answers and ranges between 0 and 35.

### Perceived professional confidence about delirium

In addition to the DKQ, a three-item questionnaire developed by the authors was also administered. These items asked the student to rate their confidence, using Likert scale items, about recognising delirium, managing delirium and providing an explanation about delirium to a patient’s family member. Participants were asked to select one of four options for each question; not at all confident, slightly confident, very confident and extremely confident. The specific items of this questionnaire were chosen by people living with delirium, specialist delirium nurses and nurse academics as being important outcome measures. Face validity was tested with 6 nursing students, who were not part of the larger sample, prior to administration.

### Ethics

This study received ethical approval in March 2019 by Queen’s University Belfast, School of Nursing and Midwifery Research Ethics Committee (Ref: G.MITCHELL.03.19.M1.V1). Participants did not provide verbal or written consent but were informed that they were under no obligation to complete any of the questionnaires. Participants gave their consent to complete the questionnaire when they actively accessed the survey web links.

### Consent/recruitment

All students (*n* = 313) received information about this study by a person unrelated to the study 1 week in advance of the workshop during a timetabled lecture and via email. Students were informed that their participation was voluntary and that they could refuse to participate in the study but were still required to attend as part of their course work. The questionnaire was administered to students in-class, via the Socrative Mobile Application, prior to the workshop and immediately afterwards. A total of 206 students completed both the pre and post-test DKQ, giving a response rate of 65.61%. Data was anonymous because students did not provide any personal or demographic information in their responses. Due to the nature of investigation, data collection focused solely on the true/false answers provided in the questionnaire. Students did not have to sign written consent forms for this workshop evaluation but were informed that they were under no obligation to complete the questionnaire. It was assumed that students gave their consent to complete the questionnaire when they actively accessed the web-link provided during class. Student participants were required to use their own laptop, computer tablet or mobile phone to complete the survey. Students, who were unable or did not wish to complete the questionnaire online, were offered a paper copy of the DKQ.

### Data analysis

Descriptive statistics were used to examine the overall pre-test and post-test scores of both the DKQ and perceived professional confidence questionnaire to ascertain the overall effect of the delirium workshop. Analysis was undertaken to illustrate pre and post-test differences across the three main question categories of the DKQ which related to presentation of delirium, risk factors of delirium and management of delirium, and at an individual level for each of the 35 items. The difference between the mean scores pre and post-test were calculated to highlight the extent of knowledge improvement post-intervention and paired t-tests were used, in both questionnaires, to establish statistical significance (*P* < 0.05).

## Results

The DKQ was completed by 206 nursing students before and after the delirium education workshop. The scores were normally distributed around the mean, with students scoring 71.33% pre-test and 81.33% post-test. This represented an increase of 10.00% overall. The standard deviation was 2.14 pre-test and 1.93 post-test. These findings were statistically significant following paired t-tests (*P* < 0.01). Descriptive statistics from the DKQ can be viewed in Table 2.

**Table 2:** Descriptive Statistics of Delirium Knowledge Questionnaire (DKQ) in Nursing Students Pre/Post-Test (*n* = 206)

DKQ Item	Pre-Test Score (% Correct Answers)	Post-Test Score (% Correct Answers)	+/− Difference (% Correct Answers)
**A. Items related to knowledge about the presentation, symptoms and outcomes of delirium**			
1. Fluctuation between orientation and disorientation is a typical feature of delirium	92.57%	94.77%	+ 2.20%
2. Symptoms of depression may mimic delirium	66.29%	82.56%	+ 16.27%
3. Patients never remember episodes of delirium	70.29%	83.14%	+ 12.85%
4. Delirium never lasts for more than a few hours	87.43%	96.51%	+ 9.08%
5. A patient who is lethargic and difficult to rouse does certainly not have a delirium	90.86%	93.60%	+ 2.74%
6. Patients with delirium are always physically and/or verbally aggressive	96.57%	95.35%	- 1.19%
7. Patients with delirium have a higher mortality rate	53.14%	68.02%	+ 14.88%
8. Behavioural changes in the course of the day are typical of delirium	90.29%	97.67%	+ 7.38%
9. A patient with delirium is likely to be easily distracted and/or have difficulty following a conversation	92.00%	95.93%	+ 3.93%
10. Patients with delirium will often experience perceptual disturbances (e.g. visual and/or auditory hallucinations)	91.43%	96.51%	+ 5.08%
***Section A Overall Score***	**83.09%**	**90.41%**	**+ 7.32%**
**B. Items related to knowledge about causes and risk factors of delirium**			
11. A patient admitted with pneumonia and having diabetes, visual and auditory disturbances has the same risk for delirium as a patient admitted with pneumonia without co-morbidities.	62.86%	63.95%	+ 1.09%
12. The risk for delirium increases with age	74.29%	95.93%	+ 21.64%
13. A patient with impaired vision is at increased risk of delirium	66.29%	92.57%	+ 26.28%
14. The greater the number of medications a patient is taking, the greater their risk of delirium	47.43%	87.56%	+ 40.13%
15. A urinary catheter reduces the risk of delirium	73.14%	93.71%	+ 20.57%
16. Poor nutrition increases the risk of delirium	72.57%	93.60%	+ 21.03%
17. Dementia is an important risk factor for delirium	74.86%	86.63%	+ 11.77%
18. Diabetes is an important risk factor for delirium	40.00%	89.53%	+ 49.53%
19. Dehydration can be a risk factor for delirium	91.43%	96.51%	+ 5.08%
20. Delirium is generally caused by alcohol withdrawal	79.43%	76.74%	- 2.69%
21. A family history of dementia predisposes a patient to delirium	50.29%	52.91%	+ 2.62%
***Section B Overall Score***	**66.60%**	**84.51%**	**+ 17.91%**
**Items related to knowledge about delirium prevention and management strategies**			
22. Treatment of delirium always includes sedation	92.00%	94.77%	+ 2.77%
23. Daily use of the Mini-Mental State Examination (MMSE) is the best way for diagnosing delirium	18.29%	37.79%	+ 19.5%
24. Providing as much staff as possible to take care at the patients’ bedside is an important strategy in the prevention of delirium	57.14%	60.47%	+ 3.3%
25. The use of physical restraints in patients at risk for delirium is the best way to ensure their safety	92.57%	91.86%	- 0.71%
26. Encouraging patients to (correctly) wear their visual/hearing aids is necessary to prevent delirium	53.14%	61.05%	+ 7.91%
27. Adequate hydration is an important strategy in the prevention of delirium	90.86%	96.51%	+ 5.65
28. The maintenance of a normal sleep-wake cycle (e.g., avoidance of sleep interruption) is an important strategy in the prevention of delirium	88.57%	94.19%	+ 5.62%
29. The use of haloperidol in preoperative surgical fracture patients is a way to prevent delirium	44.57%	59.88%	+ 15.31%
30. The stimulation of patients to perform different activities at the same time is a way to prevent delirium	47.43%	31.40%	- 16.03%
31. Keeping instructions for patients as simple as possible is important in the prevention of delirium	77.14%	81.98%	+ 4.84%
32. Early activation/ambulation (e.g., getting patients out of bed as soon as possible) of patients is an important strategy in the prevention of delirium	44.57%	63.37%	+ 18.80%
33. Providing patients with familiar objects (e.g., photos, clock, newspaper) is important to prevent sensory deprivation	93.71%	93.02%	- 0.69%
34. Avoid eye contact in the prevention of delirium because it can be seen as a threat	83.43%	85.47%	+ 2.04%
35. Keeping oral contact with the patient is an important strategy in the prevention of delirium	69.14%	80.81%	+ 11.67%
***Section C Overall Score***	**68.04%**	**73.76%**	**+ 5.72%**
**Student’s Overall Average Score (*n*** **= 206)**	**71.89% (25.16)**	**81.89% (28.66)**	**+ 10.00% (3.50)**

**Fig. 1:**
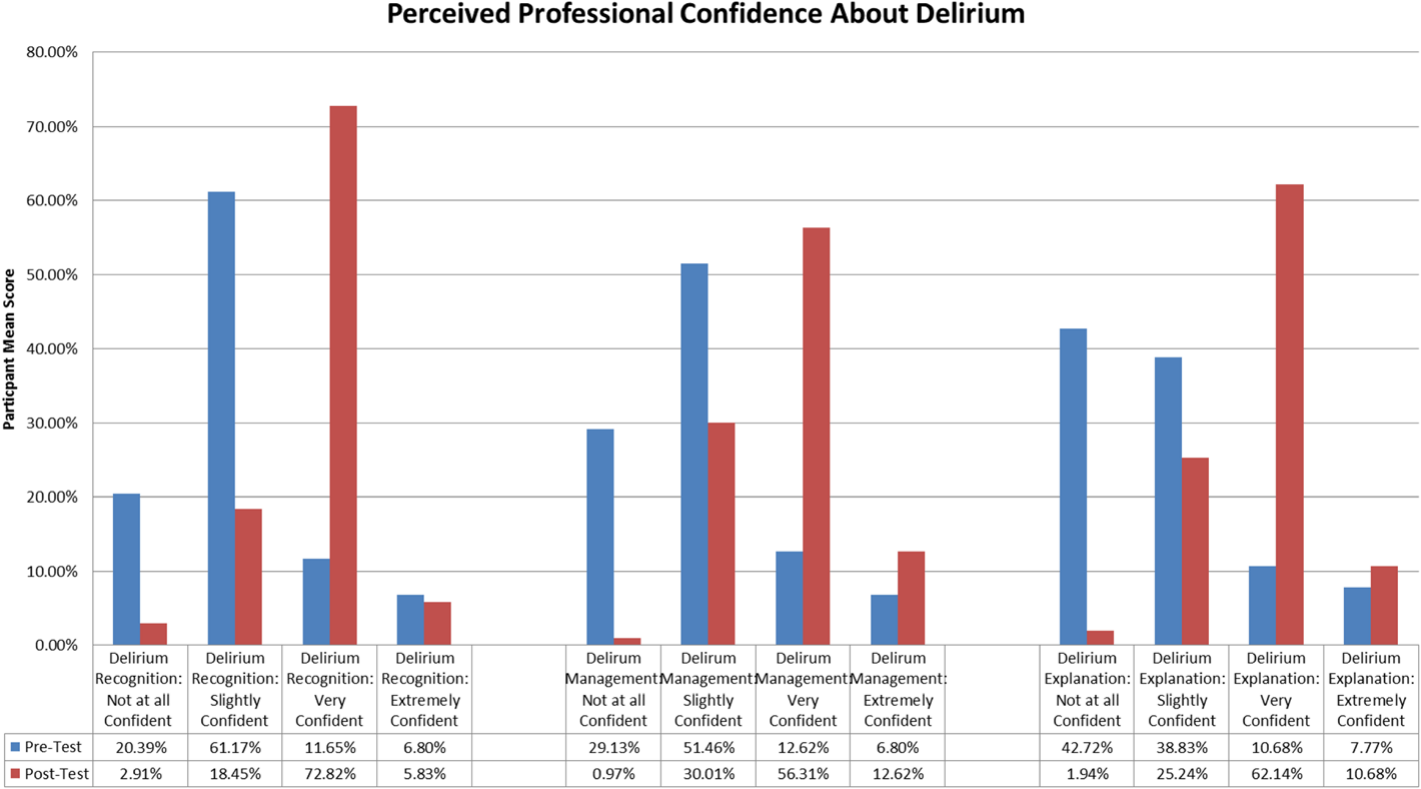
Perceived Professional Confidence about Deliriumin Nursing Students Pre/Post Test (*n* = 206)

Following the DKQ, 206 nursing students were also asked to evaluate their own perceived professional confidence about delirium. Descriptive statistics from this additional short questionnaire can be found in Fig. 1.

### Knowledge about delirium

The first part of the DKQ focused on knowledge about symptoms of delirium and this answered favourably by the students with a mean score of 83.09% recorded for this section pre-test. The most poorly answered questions in this section related to student knowledge about the impact of delirium on mortality rate and its possible similarity in presentation to depression. Post-test, students’ knowledge about symptoms of delirium improved 7.32% with students scoring an average of 90.41% (*P* < 0.01).

Section B of the DKQ focused on the causes of delirium. Pre-intervention, students scored an average of 66.60% in this section. The most poorly answered questions related to the impact of polypharmacy on delirium and the possibility of diabetes or dementia being a risk factor for developing a delirium. Following the intervention, students’ knowledge about causes of delirium increased by 17.91% with students scoring an average of 84.51% (*P* < 0.01). Large changes in knowledge were identified in questions linked to delirium risk factors associated with age (21.4% increase), vision (26.28% increase), polypharmacy (40.13% increase), presence of a urinary catheter (20.51% increase) and poor nutrition (21.03% increase) (Table 2).

The final section of the DKQ, section C, focused on delirium prevention and management. Pre-intervention, students scored an average of 68.04% in this section. The most poorly answered questions were linked to the diagnostic tools, importance of visual or hearing aids, the importance of meaningful activity and the importance of mobilisation. Following the intervention, students’ knowledge improved by 5.72%, with students’ scoring an average of 73.76% in the section (Table 2) (*P* < 0.01).

Student knowledge improvement was captured across 30/35 questions. For 4 of the 35 items, student knowledge about delirium was recorded as poorer post intervention but the difference of these findings were not statistically significant (item 6, aggression: *P* = 0.13, item 20, alcohol: *P* = 0.08, item 25, restraint: *P* = 0.31 and item 30, sensory deprivation: *P* = 0.40). Only one item in the DKQ was answered much poorer post intervention and this was related to the recommendation that patients with delirium should carry out multiple activities at the same time to combat delirium, 31.40% of respondents answered this correctly, this was significant (*P* = 0.04).

### Perceived professional confidence about delirium

Nursing students perceived professional confidence about delirium increased post-intervention (Fig. 1). 18.44% of respondents stated they felt ‘very’ or ‘extremely’ confident pre-intervention recognising delirium, which increased to 81.56% of students’ post-intervention. This represented a 60.2% increase in student perceived confidence post intervention and was statistically significant (*P* = 0.02). Similarly, in managing delirium in practice 19.41% students felt confident pre intervention with 68.93% stating they were ‘very’ or ‘extremely’ confident post-intervention. This represented an increase of 49.51% and this was significant (*P* < 0.01). The third item, explanation of delirium to a patient’s family, improved by 45.04% overall, with an 18.45% confidence pre-workshop and a 72.82% confidence post-intervention. This finding was also statistically significant (*P* < 0.01).

Overall, this intervention demonstrates a statistically significant increase in student knowledge and increasing levels of confidence in caring for people experiencing delirium.

## Discussion

Co-production in education has been associated with significant improvements in patient outcomes and is increasingly common in healthcare service and educational design [30–32]. Patients that have been involved in co-production activities also often exhibit increased levels of health literacy and empowerment [33]. Co-production is an innovative way of designing education for nursing students as it enables patients, with their lived experience, to identify priorities in partnership with clinicians and educators.

In this study, statistically significant improvements were recorded in nursing student knowledge about delirium post-intervention. This was reflected across all three question categories; symptoms of delirium, risk factors of delirium and management of delirium. In the 35-item DKQ [28], nursing student knowledge improved in 30 items. In 4 of the remaining items, nursing students scored marginally poorer post-intervention and this was not statistically significant. However, in one DKQ question about stimulation of the delirium patient, students scored worse post-intervention and this was statistically significant. Following student feedback, it was noted that the likely rationale for this misunderstanding related confusion about cognitive stimulation in delirium. Student feedback indicated that this was due to an emphasis on helping people at risk of developing delirium to maintain cognitive function through non-complex meaningful activity. Nursing students did not feel that facilitators clearly articulated that multiple activities are not recommended for a patient experiencing delirium. As a result of this feedback, facilitators of this workshop have been advised to highlight that while cognitive stimulation is important in preventing delirium, over-stimulation of a person experiencing delirium can be detrimental.

On the whole, the present study demonstrated that a co-produced education package on delirium can improve nursing student knowledge about delirium with the potential to increase their confidence. Pre-registration nursing students scored favourably in their DKQ with an overall score of 71.33% pre-intervention and 81.33% post-test. The results of this study are consistent with previous research with qualified healthcare professionals [34–36]. In a pre and post-study about the impact of a blended delirium educational package on healthcare professionals it was found that a baseline score for 59 healthcare workers, which comprised registered nurses, physiotherapists and occupational therapists, was 80.86% [28]. This represented a statistically significant (*p* < 0.01) knowledge increase of 9.71% [28] which is comparable to the current study which noted a 10.00% (*p* < 0.001) increase in knowledge post-intervention. This indicates that first year nursing students receiving a coproduced educational package, may have similar knowledge about delirium when compared to qualified healthcare professionals receiving professional development on delirium.

With regards to healthcare student education, there is limited evidence examining delirium education for undergraduate nursing students. However, research conducted with medical students highlights the importance of tailored delirium education and the positive impact this can have with trainee doctors [37–40]. In a recent UK study about delirium in undergraduate medical education, it was highlighted that 58% (*n* = 18) of medical schools in the UK had at least one learning outcome mapped to delirium yet only 4 of these medical schools evaluated their delirium education [38]. This illustrates that improving the prevention, identification and management of delirium is a complex task. Educational interventions are thought to be most effective when formal teaching is interactive and allows learners opportunities to reflect throughout, and therefore translate theory into their practice [16]. Furthermore, in relation to curriculum design, a modified Delphi process conducted with delirium experts recommended patient involvement in the production and design of any medical educational intervention about delirium [37]. The present study demonstrated a novel approach to undergraduate nursing delirium education through the coproduction of the educational intervention.

### Strengths, limitations and future research

The novel approach to collaborative co-production in designing this learning package in undergraduate nursing education about delirium is a strength of this study. Further, this intervention led to significant improvements in student nurse knowledge about delirium post-intervention and this has the potential to support participants in the timely recognition and management of delirium. Finally, this study reports on a high number of participants (*n* = 206) at the same stage of their nurse training (year one students with 12 weeks clinical placement experience). Although a large cohort, generalisability of the findings may be limited at this point as the sample was from one cohort of nursing students in one university in Northern Ireland. This study also did not collect any demographic data from nursing students which could have detailed previous care experience of participants. This data may would have enhanced data analysis and further explain changes that occurred after the workshop. A recommendation for future research is to conduct a similar intervention for different cohorts of pre-registration nursing students across multiple universities. To ascertain if students retain this knowledge, a longitudinal follow up of nursing students on placement is recommended. Furthermore, qualitative research is needed to understand how student nurses apply their knowledge about delirium during their clinical placements. With regards to the mode of education, it is also worth considering how high-fidelity simulation could further enhance delirium education for nursing students. The co-production methodology would be very appropriate for supporting the development of student learning scenarios.

## Conclusion

This research highlights how co-produced education about delirium can enhance knowledge and perceived clinical confidence. This intervention supported nursing students in increasing their knowledge regarding the prevention, identification and management of delirium in primary, secondary and tertiary care. Students also perceived improvements with regards to their confidence recognising, managing and explaining delirium. The lack of literature in this area suggests an urgent need to improve delirium knowledge in healthcare education to support future practitioners to more effectively support patients experiencing delirium.

To date, this co-produced education resource has been used in more than 50 clinical areas and universities across the United Kingdom. Continued delirium education is important for nursing students due to their role in prevention, recognition and management of the condition. This co-produced workshop provides a good introduction to delirium from the perspective of people with lived experience. Further continuing education in year two or year three of the nursing programme, possibly in the form of high-fidelity simulation using co-production, would support further support nursing students in translating this important knowledge to practice.

## Supplementary information

**Additional file 1.** Download copy of the co-produced delirium education resource (supplementary file 1).

## Data Availability

The datasets generated and/or analysed during the current study are not publicly available as per our ethical approval but are available from the corresponding author on reasonable request.

## Abbreviations

DKQ: Delirium Knowledge Questionnaire
QUB: Queen’s University Belfast
RCN: Royal College of Nursing
4AT: 4 A’s Test: A Rapid Clinical Test for Delirium

## References

[1] World Health Organization (2004). ICD-10: international statistical classification of diseases and related health problems: tenth revision.

[2] Davis D, Kreisel S, Muniz Terrera G, Hall A, Morandi A, Boustani M (2013). The epidemiology of delirium: challenges and opportunities for population studies. Am J Geriatr Psychiatry.

[3] Ospina J, King F, Madva E, Celano C (2018). Epidemiology, mechanisms, diagnosis and treatment of delirium: a narrative review. Clin Med and Ther.

[4] Saravana-Bawan B, Warkentin LM, Rucker D, Carr F, Churchill TA, Khadaroo RG (2019). Incidence and predictors of postoperative delirium in the older acute care surgery population: a prospective study. Can J Surg.

[5] Bellelli G, Morandi A, Di Santo SG, Mazzone A, Cherubini A, Mossello E (2016). Delirium day: a nationwide point prevalence study of delirium in older hospitalized patients using an easy standardized diagnostic tool. BMC Med.

[6] Ryan DJ, O'Regan NA, Caoimh RÓ, Clare J, Connor M, Leonard M (2013). Delirium in an adult acute hospital population: predictors, prevalence and detection. BMJ Open.

[7] Lange P, Lamanna M, Watson R, Maier AB (2019). Undiagnosed delirium is frequent and difficult to predict: results from a prevalence survey of a tertiary hospital. J Clin Nurs.

[8] Mitchell G. Undiagnosed delirium is common and difficult to predict amongst hospitalised patients. Evid Based Nurs. 2019. 10.1136/ebnurs-2019-103120. 10.1136/ebnurs-2019-103120 31296611

[9] Israni J, Lesser A, Kent T, Ko K (2018). Delirium as a predictor of mortality in US Medicare beneficiaries discharged from the emergency department: a national claims-level analysis up to 12 months. BMJ Open.

[10] Marcantonio ER (2017). Delirium in hospitalized older adults. New Eng J Med.

[11] Lieow JLM, Chen FSM, Song, Tang PS, Kowitlawakul Y, Mukhopadhyay A (2019). Effectiveness of an advanced practice nurse-led delirium education and training programme. Int Nurs Rev.

[12] Lee S, Fisher J, Wand A, Millsen K, Detroyer E, Sockalingham S, et al. Teodorczuk. Eur Geriatr Med. 2019. 10.1007/s41999-019-00278. 10.1007/s41999-019-00278-x 32297244

[13] Marr S, McKibbon K, Patel A, McKinnon J, Hillier L. The geriatric certificate program: collaborative partnerships for building capacity for a competent workforce. Gerontol Geriatr Educ. 2019. 10.1080/02701960.2019.1572004. 10.1080/02701960.2019.1572004 30706766

[14] Sinvani L, Kozikowski K, Pekmezaris R, Akerman M, Wolf-Klein G (2016). Delirium: a survey of healthcare professionals’ knowledge, beliefs, and practices. J Am Geriatr Soc.

[15] Hshieh T, Yue J, Oh E, Puelle M, Dowal S, Travison T (2015). Effectiveness of multicomponent nonpharmacological delirium interventions: a meta-analysis. JAMA Int Med.

[16] Yanamadala M, Wieland D, Heflin M (2013). Educational interventions to improve recognition of delirium: a systematic review. J Am Geriatr Soc.

[17] Wand A (2011). Evaluating the effectiveness of educational interventions to prevent delirium. Aust J Ageing.

[18] Coyle M, Chang H, Burns P, Traynor V (2018). Impact of interactive education on healthcare practitioners and older adults at risk of delirium: a literature review. J Gerontol Nurs.

[19] Sockalingam S, Tan A, Hawa R, Pollex H, Abbey S, Hodges BD (2014). Interprofessional education for delirium care: a systematic review. J Interprof Care.

[20] Yaghmour SM, Gholizadeh L (2016). Review of nurses’ knowledge of delirium, dementia and depressions (3ds): systematic literature review. Open J Nursing.

[21] Bleakley A, Bligh J (2008). Students learning from patients: let’s get real in medical education. Advances in health sciences education. Theory and Pract.

[22] Palumbo R (2016). Contextualizing co-production of health care: a systematic literature review. Int J Pub Sector Manag.

[23] Simons L (2016). Integrated service user-led teaching in higher education: experiences and learning points. Ment Health Rev J.

[24] Morgan A, Jones D (2009). Perceptions of service user and carer involvement in healthcare education and impact on students’ knowledge and practice: a literature review. Med Teach.

[25] Royal College of Nursing: https://www.rcn.org.uk/clinical-topics/older-people/delirium.

[26] Dementia Together Northern Ireland: http://pha.site/Deliriumvid.

[27] European Delirium Association: http://www.europeandeliriumassociation.com/patient-video.html.

[28] Detroyer E, Dobbels F, Debonnaire D, Irving K, Teodorczuk A, Fick A (2016). The effect of an interactive delirium e-learning tool on healthcare workers’ delirium recognition, knowledge and strain in caring for delirious patients: a pilot pre-test/post-test study. BMC Med Educ.

[29] Hare M, Wynaden D, McGowan S (2008). A questionnaire to determine nurses’ knowledge of delirium and its risk factors. Contemp Nurse.

[30] Hjelmfors L, Stromberg A, Friedrichsen M, Sandgren A, Martensson J, Jaarsma T (2018). Using co-design to develop an intervention to improve communication about the heart failure trajectory and end-of-life care. BMC Pall Care.

[31] Wright R, Lowton K, Robert G, Grudzen C, Grocott P (2017). Using experience-based co-design with older patients, their families and staff to improve palliative care experiences in the emergency department: a reflective critique on the process and outcomes. Int J Nurs Stud.

[32] Israilov S, Cho H (2017). Hierarchy, overwhelmed patients, and conflicts of interest in health care quality and safety. AMA J Ethics.

[33] Needham C, Carr S (2009). SCIE research briefing 31: co-production: an emerging evidence base for adult social care transformation.

[34] Fick D, Hodo D, Lawrence F, Inouye S (2007). Recognizing delirium superimposed on dementia: assessing nurses’ knowledge using case vignettes. J Gerontol Nurs.

[35] McCrow J, Sullivan K, Beattie E (2014). Delirium knowledge and recognition: a randomized controlled trial of web-based educational intervention for acute care nurses. Nurse Educ Today.

[36] van de Steeg L, Ijkema R, Langelaan M, Wagner C (2014). Can an e-learning course improve nursing care for older people at risk of delirium: a stepped wedge cluster randomised trial. BMC Geriatr.

[37] Copeland C, Fisher J, Teodorczuk A (2018). Development of an international undergraduate curriculum for delirium using a modified delphi process. Age Ageing.

[38] Fisher JM, Gordon AL, MacLullich AMJ, Tullo E, Davis DHJ, Blundell A (2015). Towards an understanding of why undergraduate teaching about delirium does not guarantee gold-standard practice—results from a UK national survey. Age Ageing.

[39] Buijs-Spanjers KR, Hegge HH, Jansen CJ, Hoogendoorn E, de Rooij SE (2018). A web-based serious game on delirium as an educational intervention for medical students: randomized controlled trial. JMIR Serious Games.

[40] Baessler F, Cipriandidis A, Rizvi AZ, Weidlich J, Wagner FL, Klein SB (2019). Delirium: medical students’ knowledge and effectiveness of different teaching methods. Am J Geriatr Psychiatry.

