# Supplementary material for: Evaluation of a co-produced delirium awareness programme for undergraduate nursing students in Northern Ireland: a pre-test/post-test study
Source: BMC Nurs. 2020 Apr 26;19:34. doi: 10.1186/s12912-020-00427-9 (PMC7183703; doi:10.1186/s12912-020-00427-9)
Supplement: Supplementary file 1 — Additional file 1. Download copy of the co-produced delirium education resource (supplementary file 1). [file 12912_2020_427_MOESM1_ESM.pptx]

## Slide 1
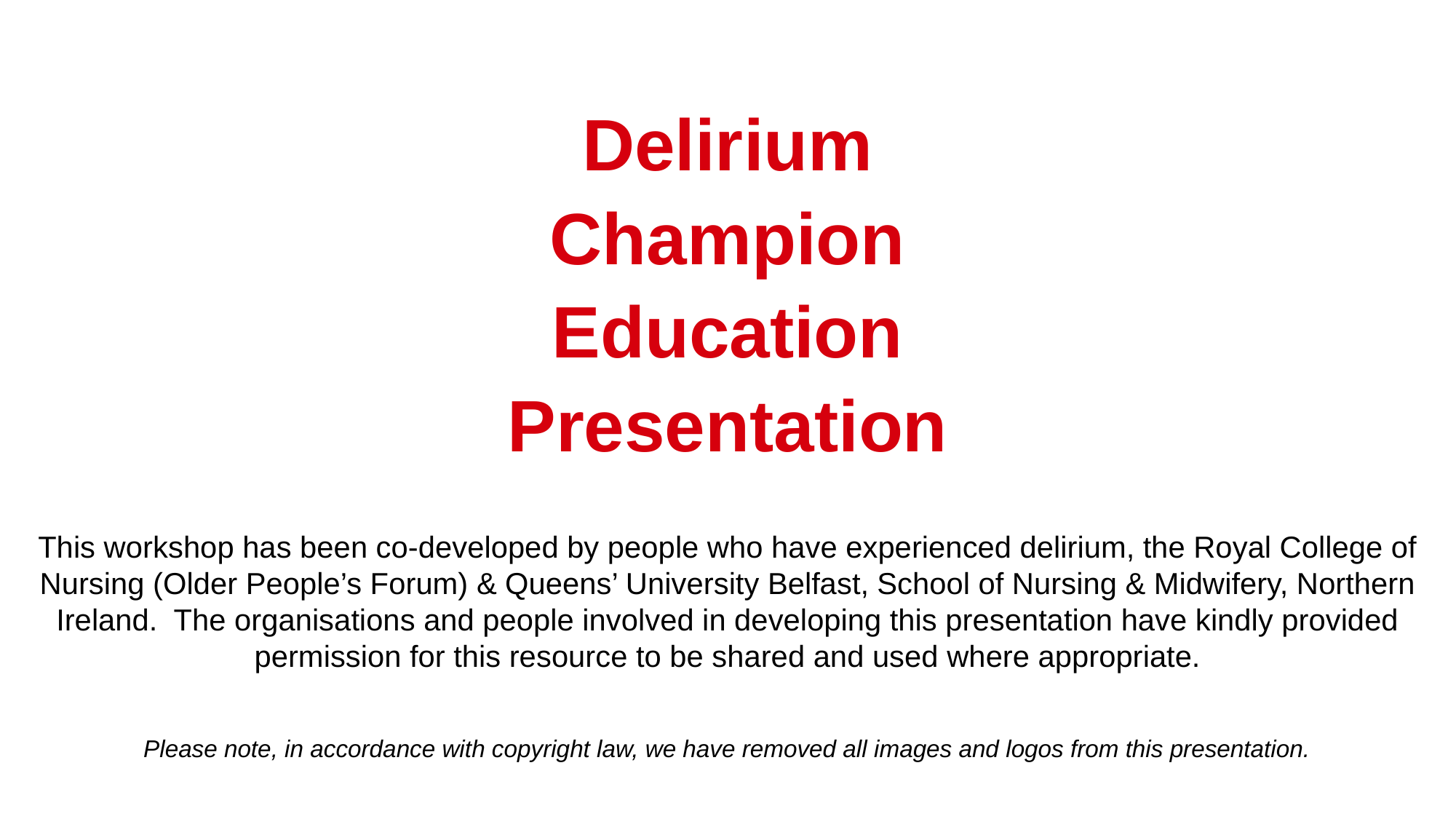

Delirium
Champion
Education
Presentation
This workshop has been co-developed by people who have experienced delirium, the Royal College of Nursing (Older People’s Forum) & Queens’ University Belfast, School of Nursing & Midwifery, Northern Ireland. The organisations and people involved in developing this presentation have kindly provided permission for this resource to be shared and used where appropriate.
Please note, in accordance with copyright law, we have removed all images and logos from this presentation.

## Slide 2
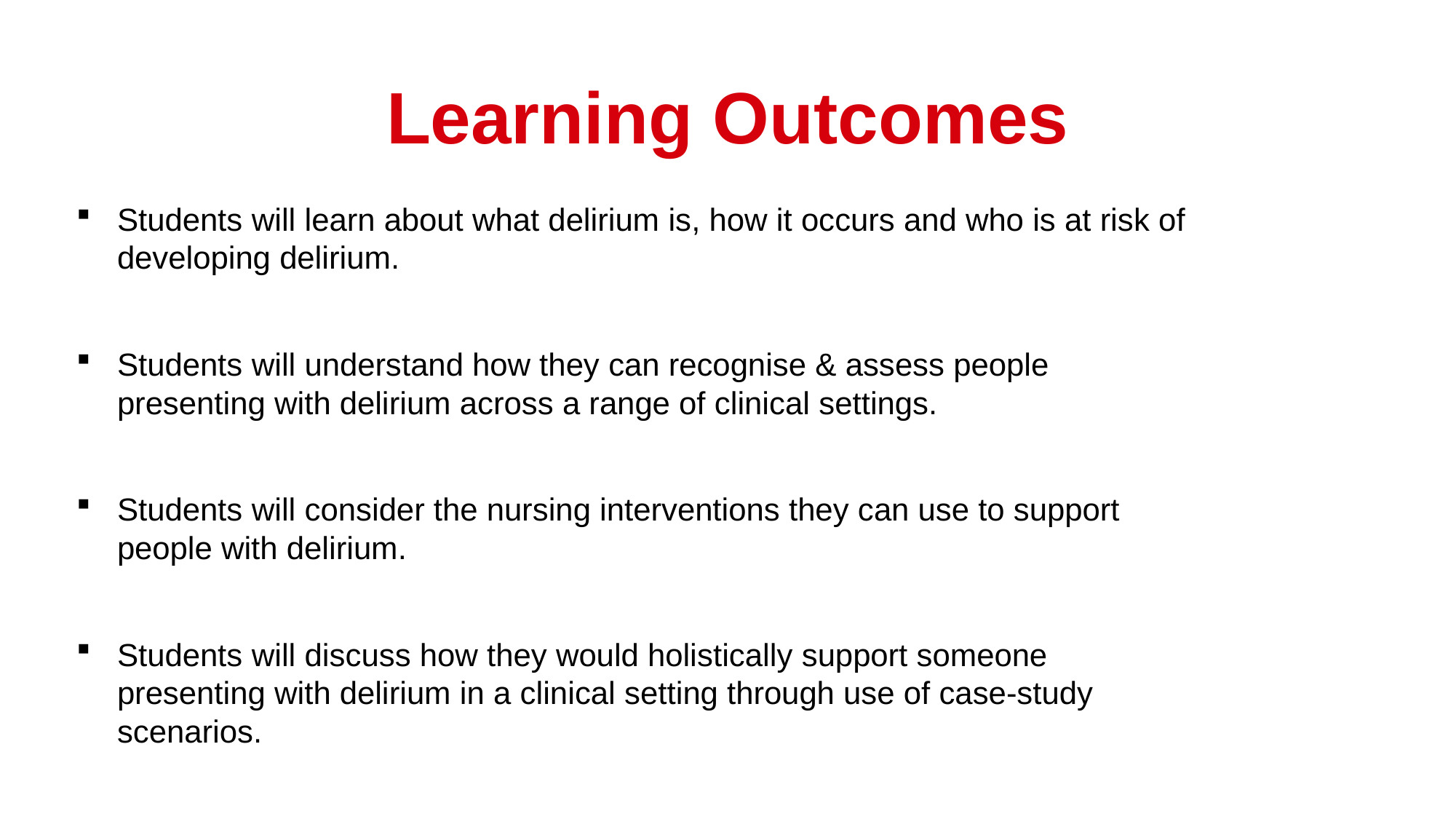

Learning Outcomes
Students will learn about what delirium is, how it occurs and who is at risk of developing delirium.
Students will understand how they can recognise & assess people presenting with delirium across a range of clinical settings.
Students will consider the nursing interventions they can use to support people with delirium.
Students will discuss how they would holistically support someone presenting with delirium in a clinical setting through use of case-study scenarios.

## Slide 3
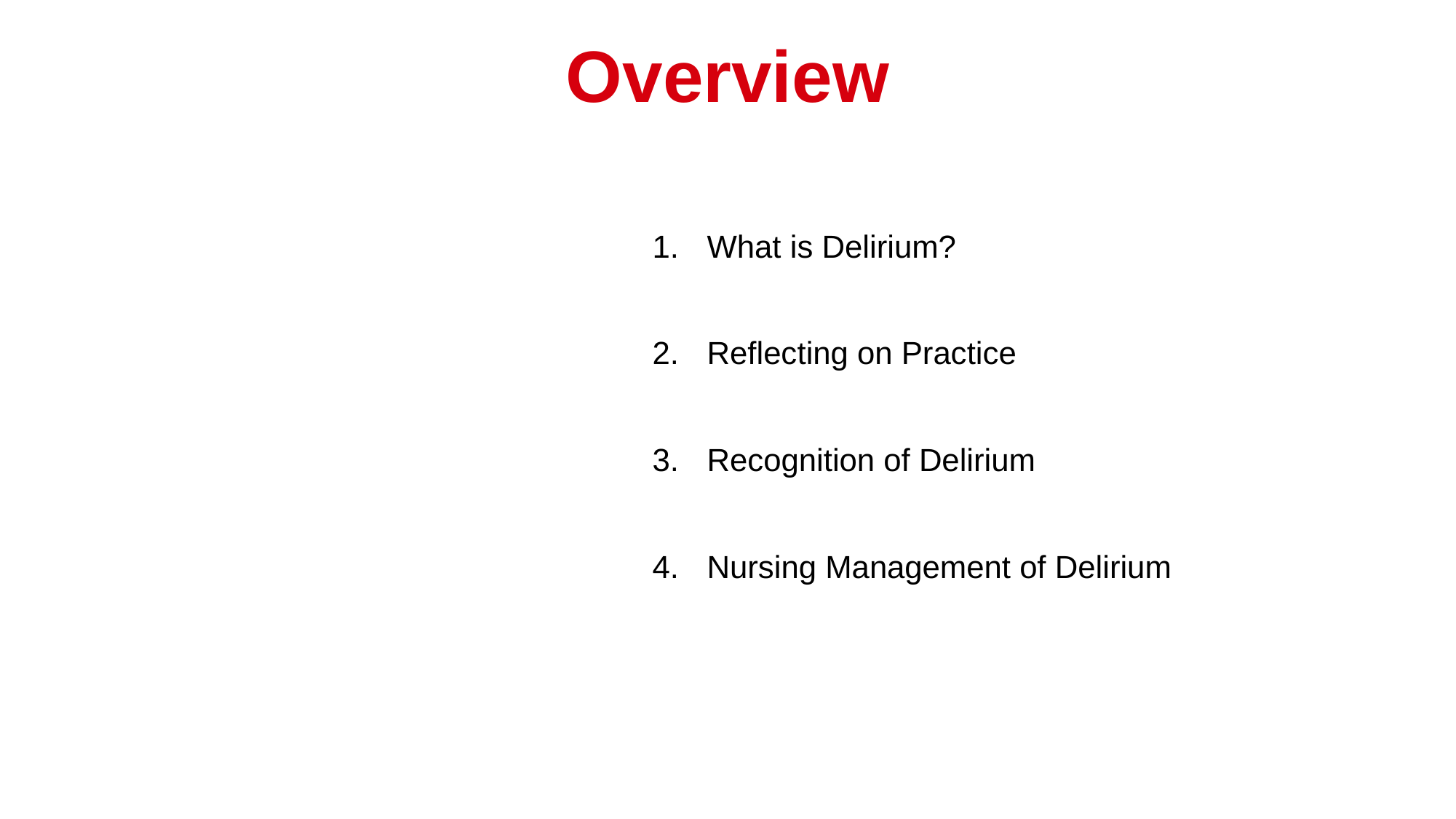

Overview
What is Delirium?
Reflecting on Practice
Recognition of Delirium
Nursing Management of Delirium

## Slide 4
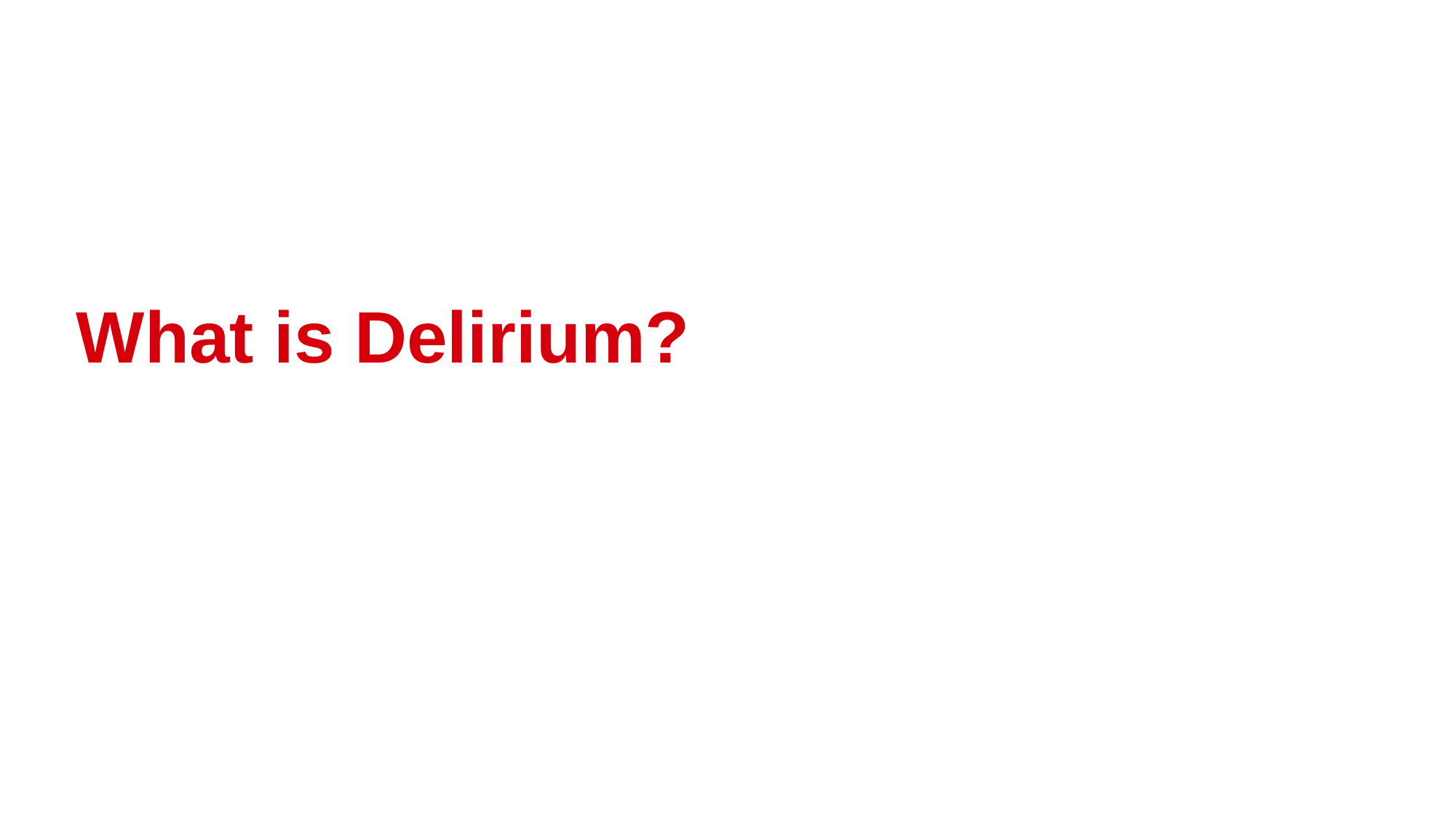

What is Delirium?

## Slide 5
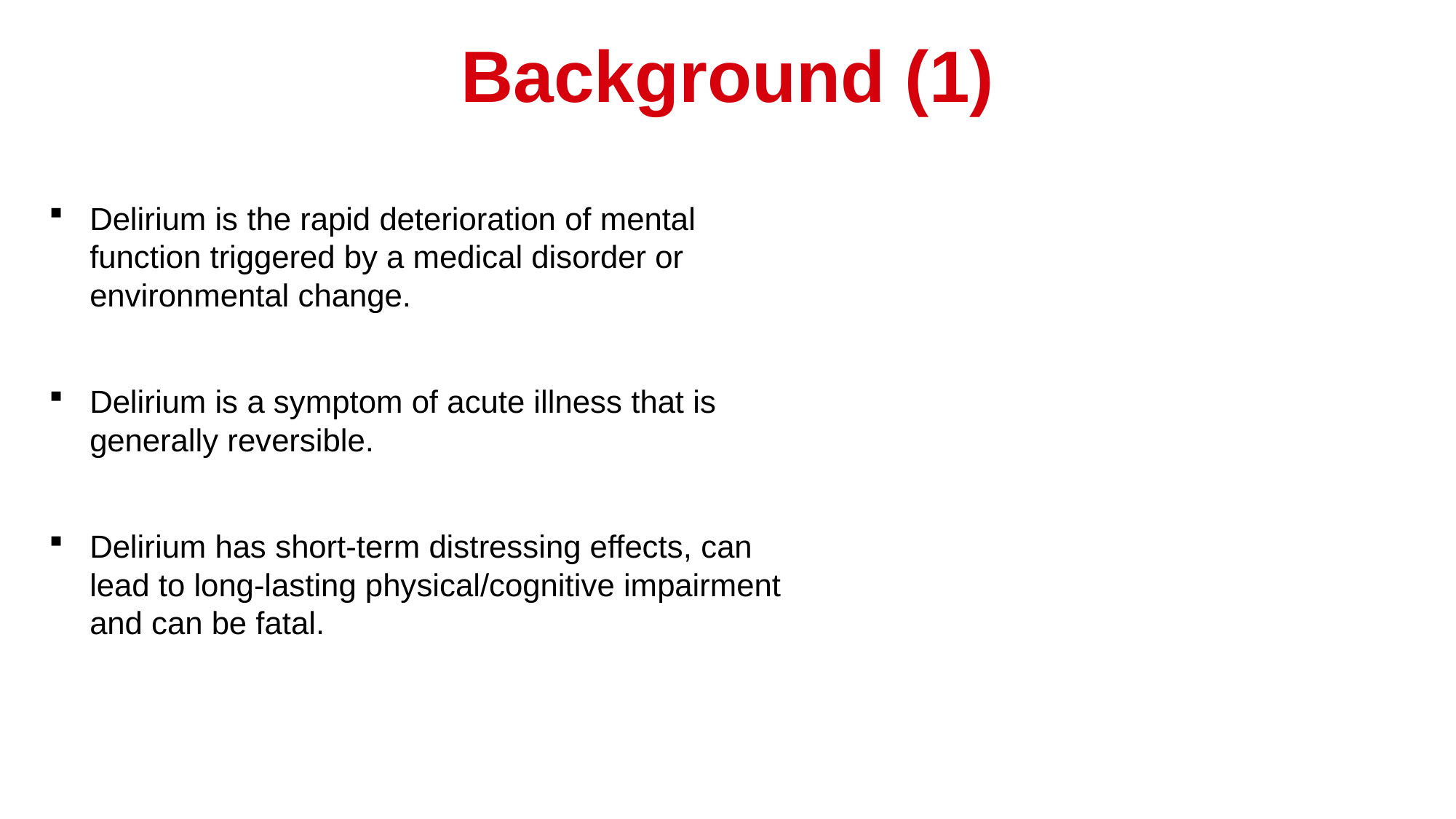

Background (1)
Delirium is the rapid deterioration of mental function triggered by a medical disorder or environmental change.
Delirium is a symptom of acute illness that is generally reversible.
Delirium has short-term distressing effects, can lead to long-lasting physical/cognitive impairment and can be fatal.

## Slide 6
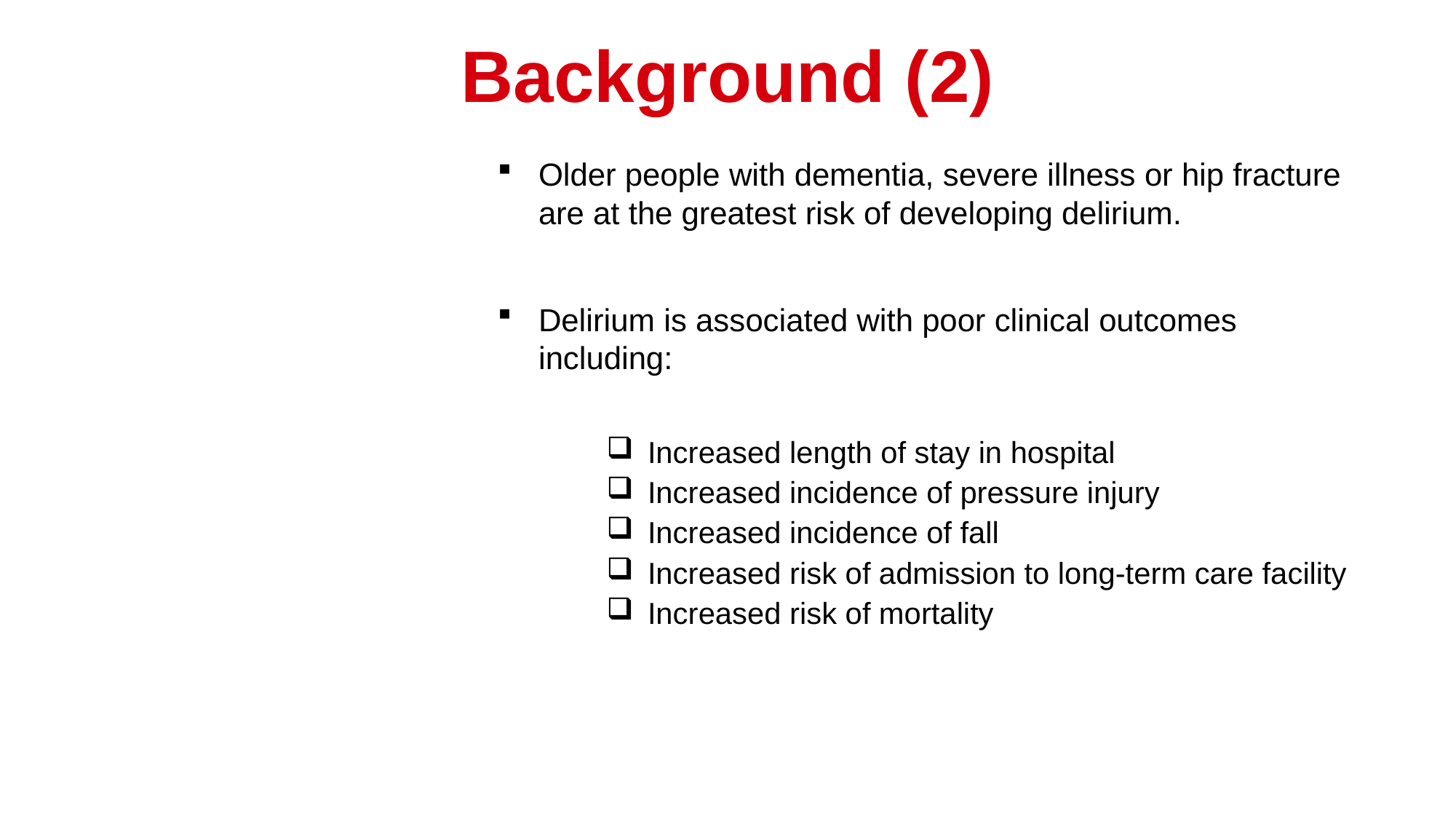

Background (2)
Older people with dementia, severe illness or hip fracture are at the greatest risk of developing delirium.
Delirium is associated with poor clinical outcomes including:
Increased length of stay in hospital
Increased incidence of pressure injury
Increased incidence of fall
Increased risk of admission to long-term care facility
Increased risk of mortality

## Slide 7
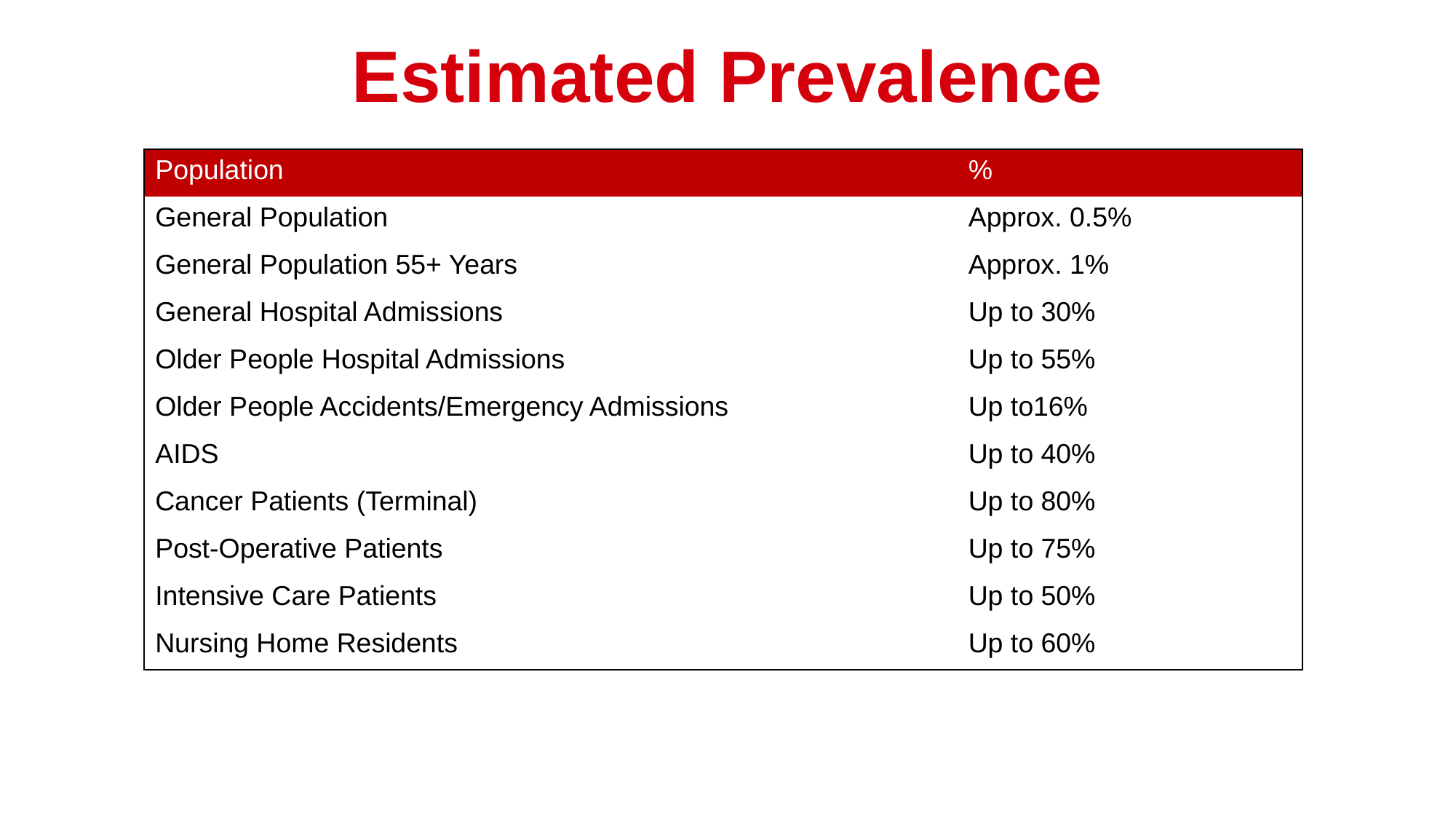

Estimated Prevalence
| Population | % |
| --- | --- |
| General Population | Approx. 0.5% |
| General Population 55+ Years | Approx. 1% |
| General Hospital Admissions | Up to 30% |
| Older People Hospital Admissions | Up to 55% |
| Older People Accidents/Emergency Admissions | Up to16% |
| AIDS | Up to 40% |
| Cancer Patients (Terminal) | Up to 80% |
| Post-Operative Patients | Up to 75% |
| Intensive Care Patients | Up to 50% |
| Nursing Home Residents | Up to 60% |

## Slide 8
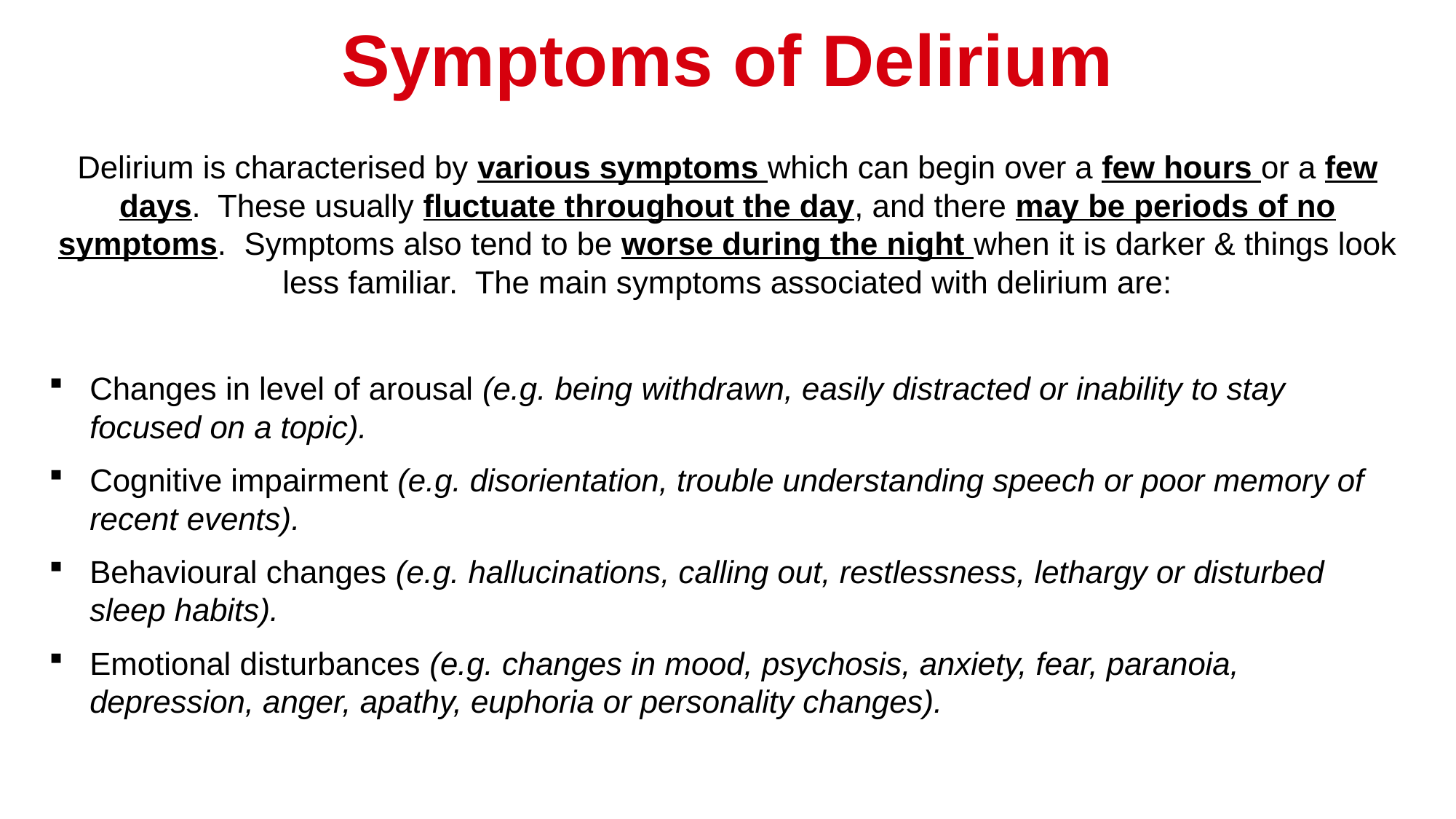

Symptoms of Delirium
Delirium is characterised by various symptoms which can begin over a few hours or a few days. These usually fluctuate throughout the day, and there may be periods of no symptoms. Symptoms also tend to be worse during the night when it is darker & things look less familiar. The main symptoms associated with delirium are:
Changes in level of arousal (e.g. being withdrawn, easily distracted or inability to stay focused on a topic).
Cognitive impairment (e.g. disorientation, trouble understanding speech or poor memory of recent events).
Behavioural changes (e.g. hallucinations, calling out, restlessness, lethargy or disturbed sleep habits).
Emotional disturbances (e.g. changes in mood, psychosis, anxiety, fear, paranoia, depression, anger, apathy, euphoria or personality changes).

## Slide 9
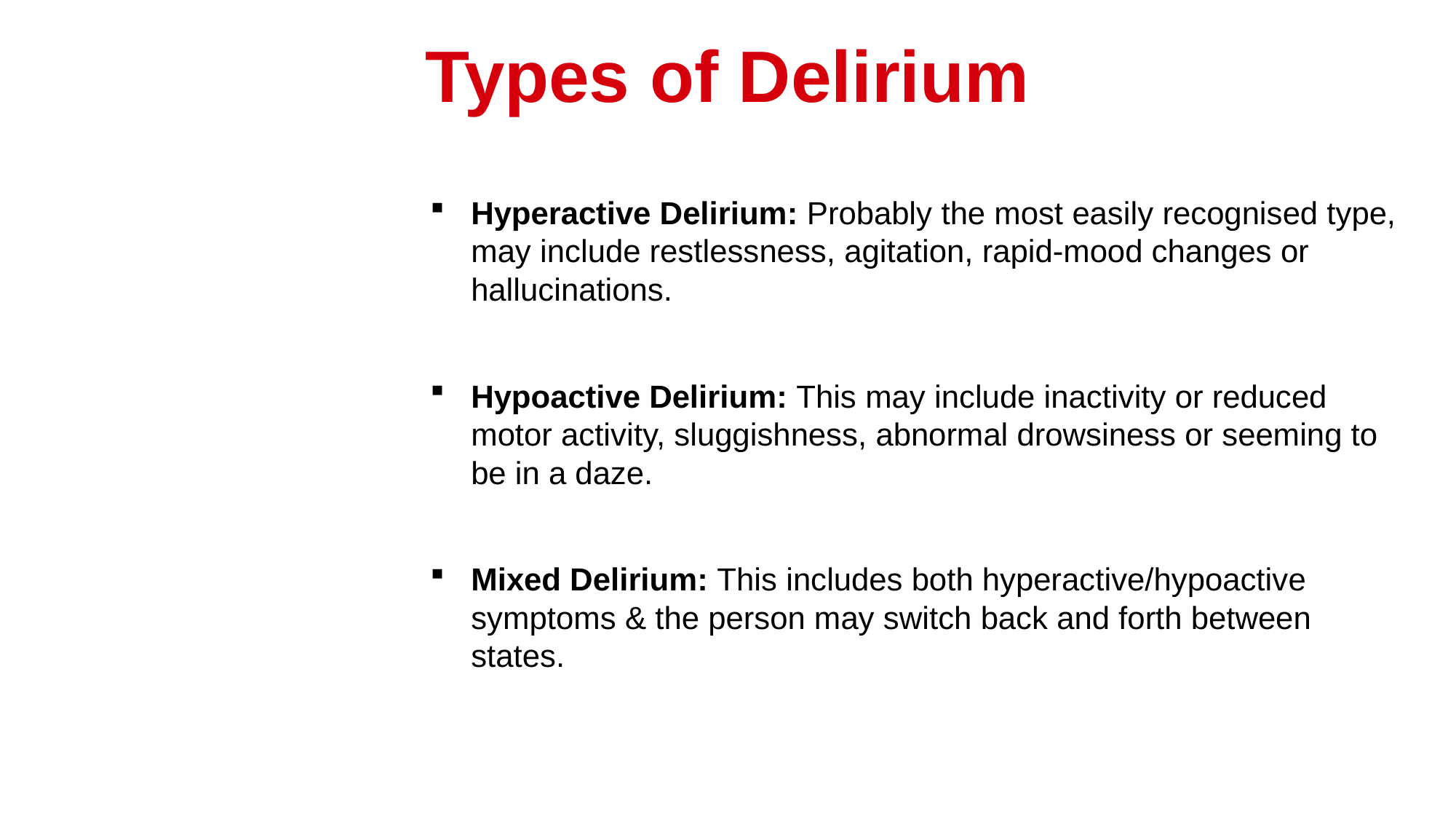

Types of Delirium
Hyperactive Delirium: Probably the most easily recognised type, may include restlessness, agitation, rapid-mood changes or hallucinations.
Hypoactive Delirium: This may include inactivity or reduced motor activity, sluggishness, abnormal drowsiness or seeming to be in a daze.
Mixed Delirium: This includes both hyperactive/hypoactive symptoms & the person may switch back and forth between states.

## Slide 10
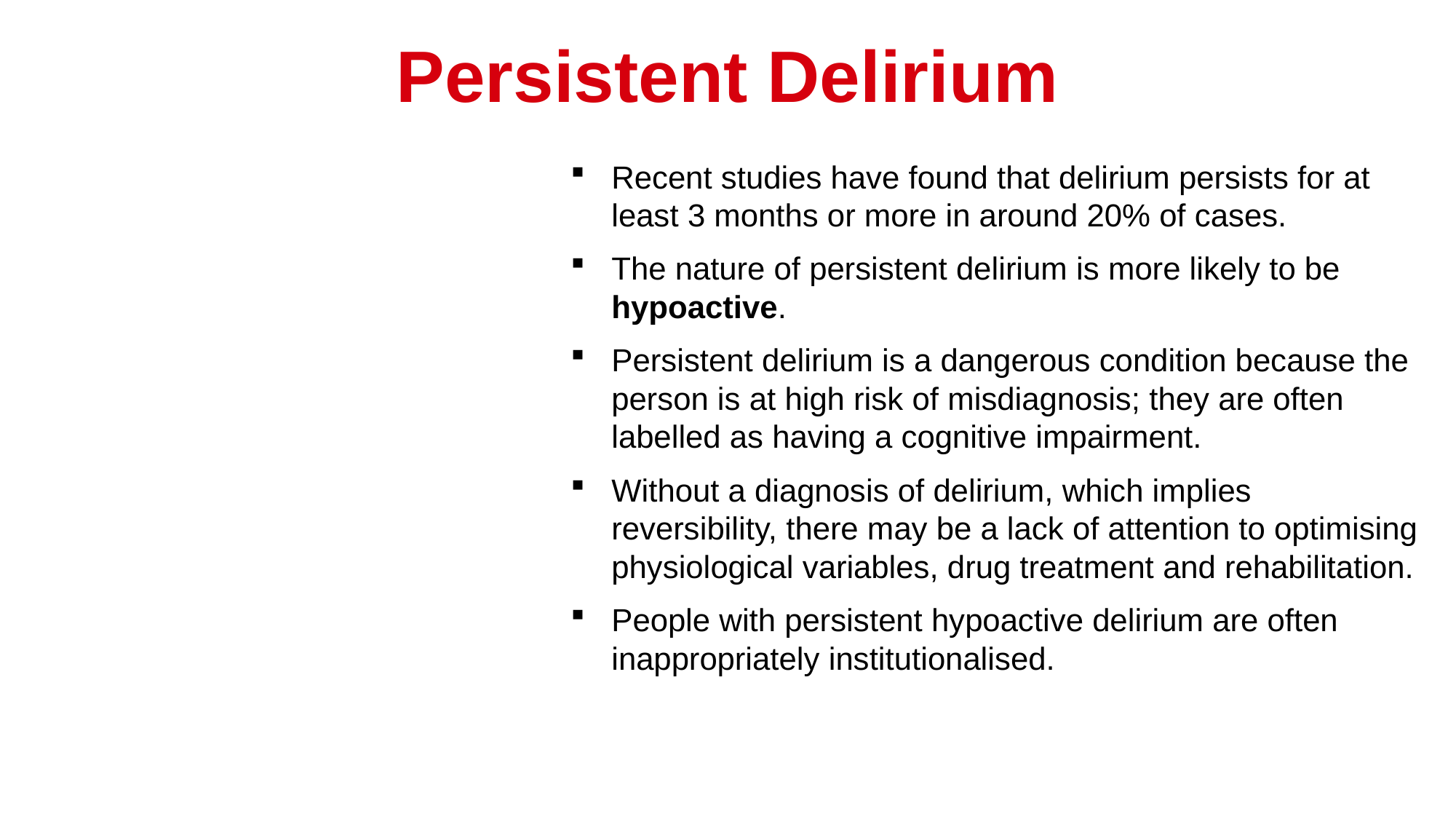

Persistent Delirium
Recent studies have found that delirium persists for at least 3 months or more in around 20% of cases.
The nature of persistent delirium is more likely to be hypoactive.
Persistent delirium is a dangerous condition because the person is at high risk of misdiagnosis; they are often labelled as having a cognitive impairment.
Without a diagnosis of delirium, which implies reversibility, there may be a lack of attention to optimising physiological variables, drug treatment and rehabilitation.
People with persistent hypoactive delirium are often inappropriately institutionalised.

## Slide 11
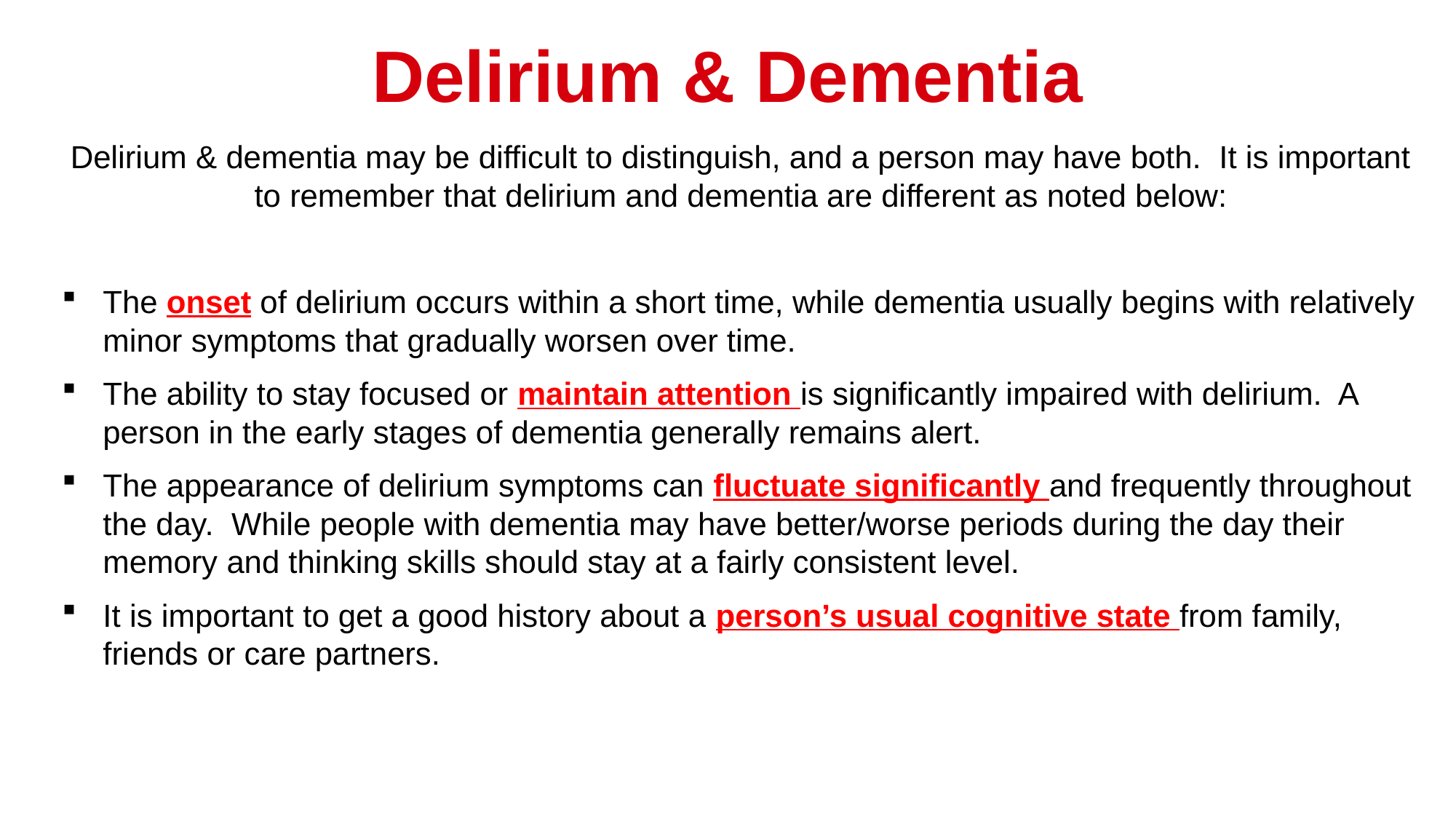

Delirium & Dementia
Delirium & dementia may be difficult to distinguish, and a person may have both. It is important to remember that delirium and dementia are different as noted below:
The onset of delirium occurs within a short time, while dementia usually begins with relatively minor symptoms that gradually worsen over time.
The ability to stay focused or maintain attention is significantly impaired with delirium. A person in the early stages of dementia generally remains alert.
The appearance of delirium symptoms can fluctuate significantly and frequently throughout the day. While people with dementia may have better/worse periods during the day their memory and thinking skills should stay at a fairly consistent level.
It is important to get a good history about a person’s usual cognitive state from family, friends or care partners.

## Slide 12
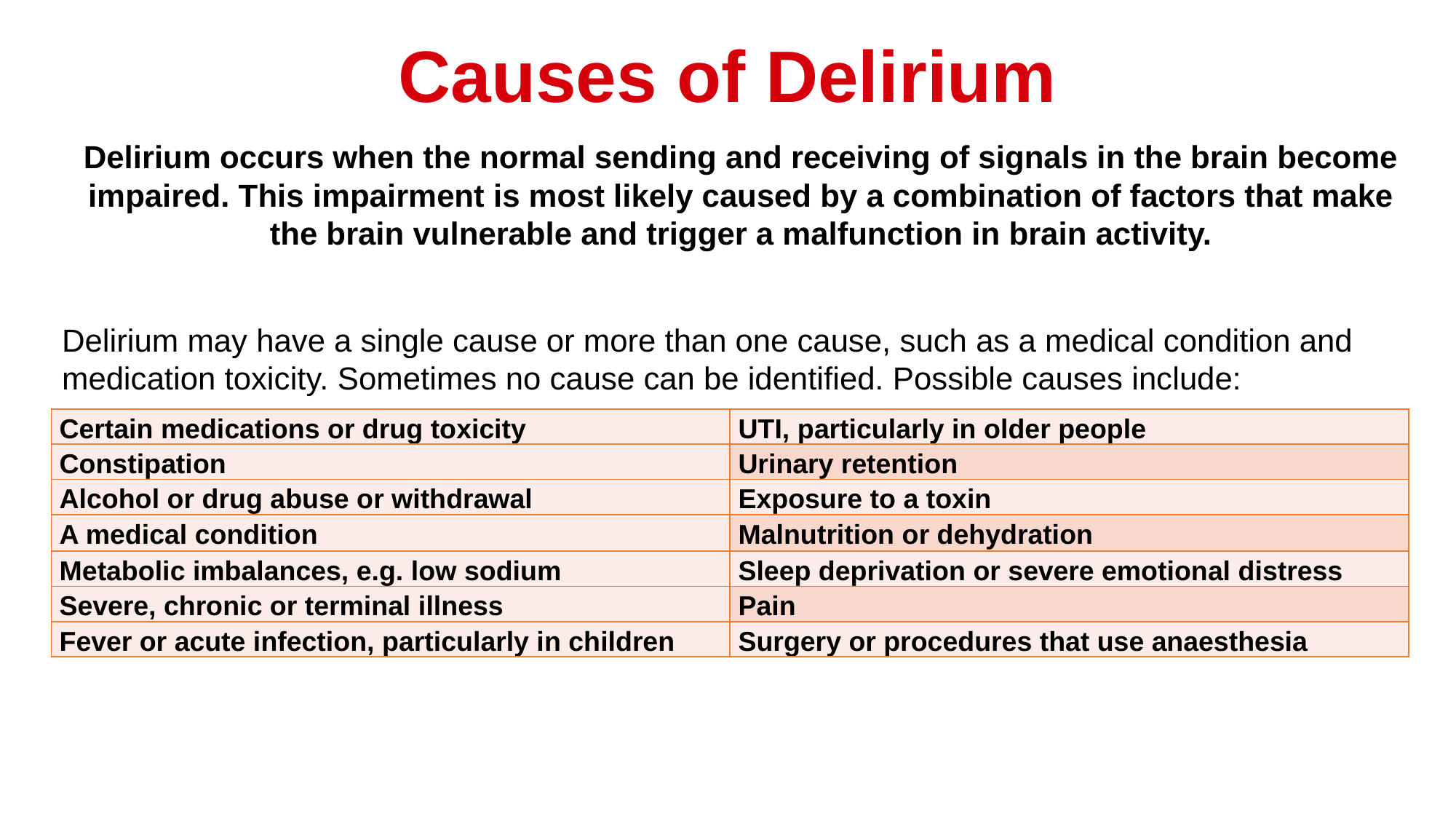

Causes of Delirium
Delirium occurs when the normal sending and receiving of signals in the brain become impaired. This impairment is most likely caused by a combination of factors that make the brain vulnerable and trigger a malfunction in brain activity.
Delirium may have a single cause or more than one cause, such as a medical condition and medication toxicity. Sometimes no cause can be identified. Possible causes include:
| Certain medications or drug toxicity | UTI, particularly in older people |
| --- | --- |
| Constipation | Urinary retention |
| Alcohol or drug abuse or withdrawal | Exposure to a toxin |
| A medical condition | Malnutrition or dehydration |
| Metabolic imbalances, e.g. low sodium | Sleep deprivation or severe emotional distress |
| Severe, chronic or terminal illness | Pain |
| Fever or acute infection, particularly in children | Surgery or procedures that use anaesthesia |

## Slide 13
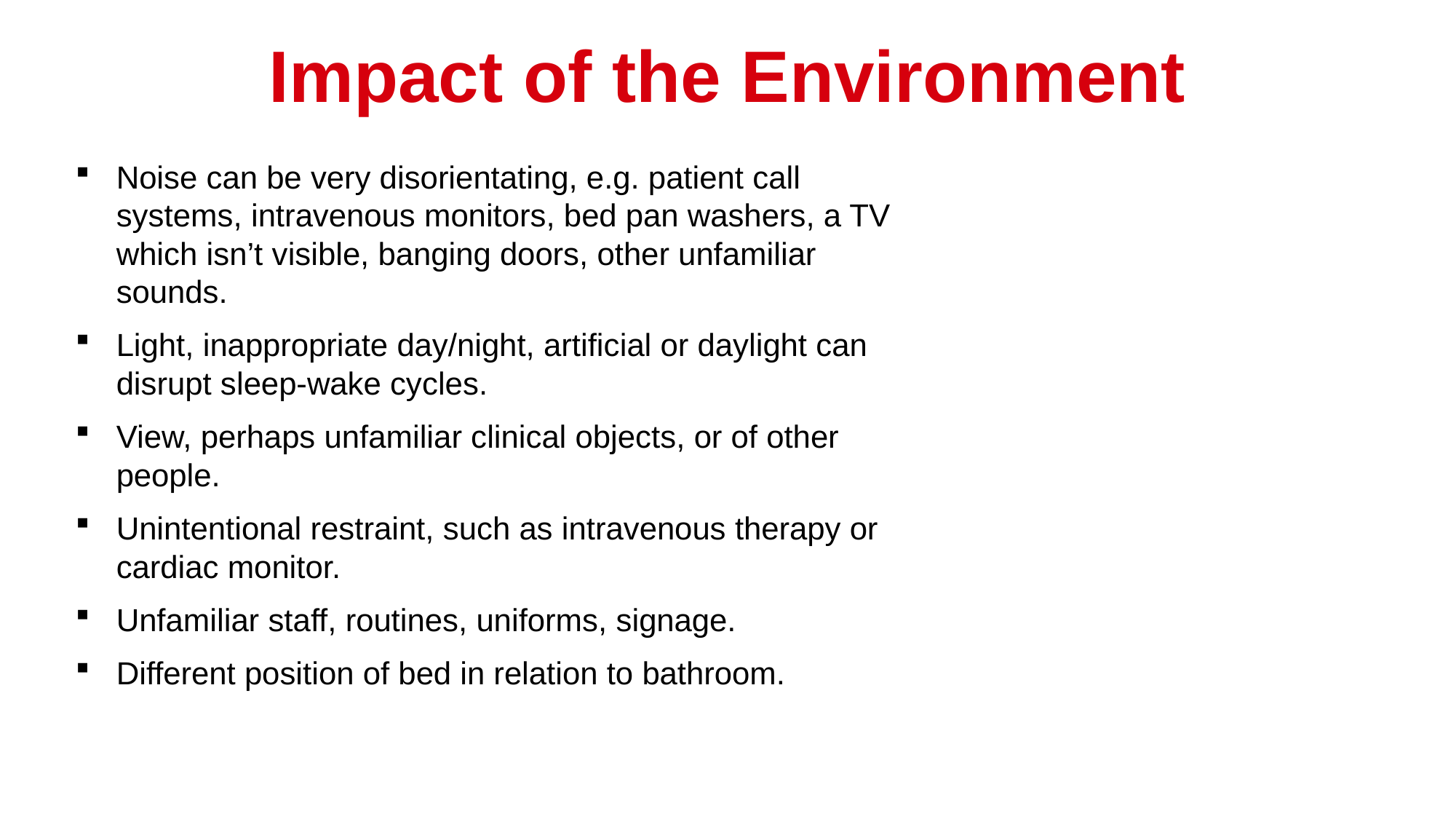

Impact of the Environment
Noise can be very disorientating, e.g. patient call systems, intravenous monitors, bed pan washers, a TV which isn’t visible, banging doors, other unfamiliar sounds.
Light, inappropriate day/night, artificial or daylight can disrupt sleep-wake cycles.
View, perhaps unfamiliar clinical objects, or of other people.
Unintentional restraint, such as intravenous therapy or cardiac monitor.
Unfamiliar staff, routines, uniforms, signage.
Different position of bed in relation to bathroom.

## Slide 14
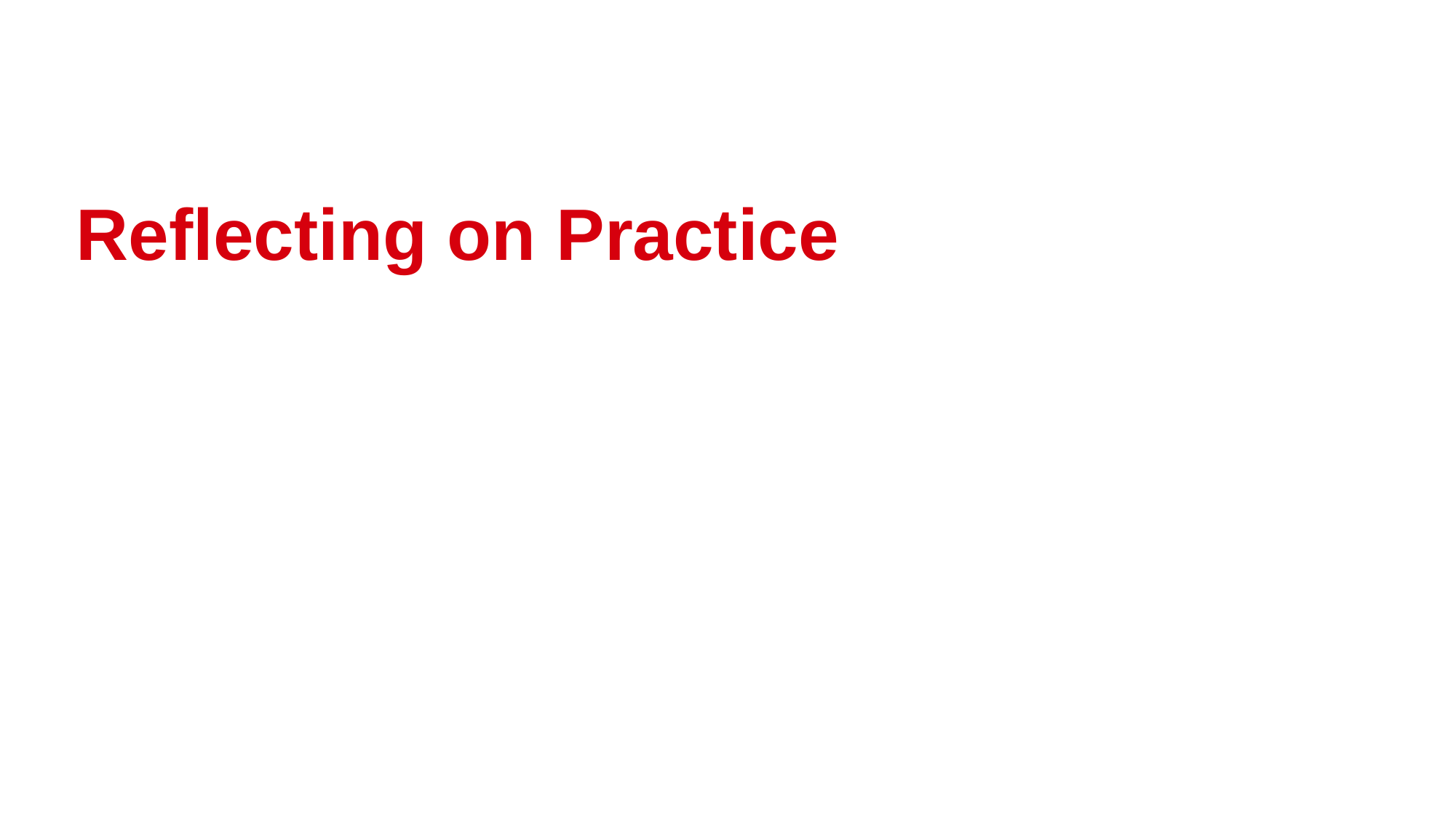

Reflecting on Practice

## Slide 15
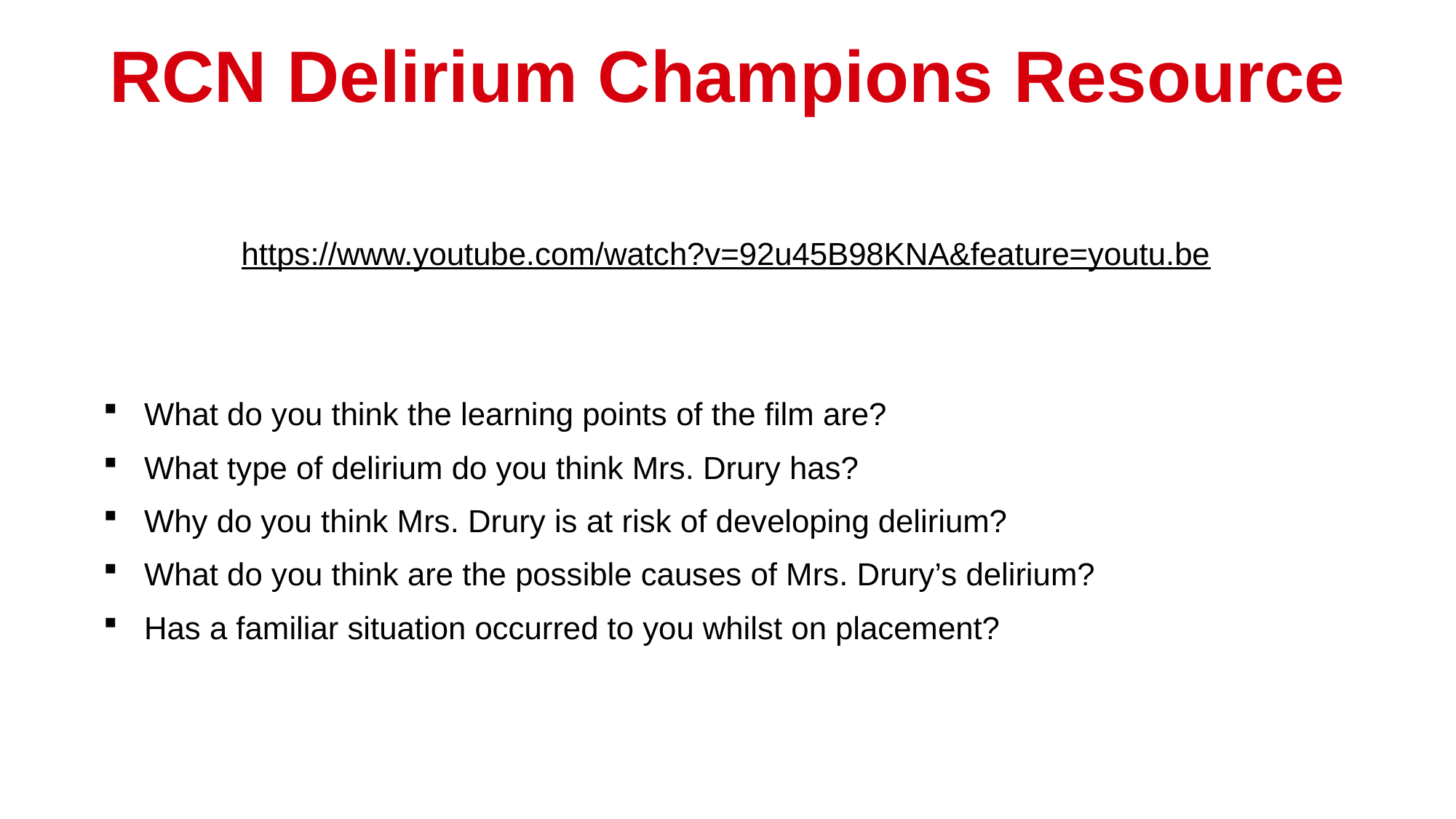

RCN Delirium Champions Resource
https://www.youtube.com/watch?v=92u45B98KNA&feature=youtu.be
What do you think the learning points of the film are?
What type of delirium do you think Mrs. Drury has?
Why do you think Mrs. Drury is at risk of developing delirium?
What do you think are the possible causes of Mrs. Drury’s delirium?
Has a familiar situation occurred to you whilst on placement?

## Slide 16
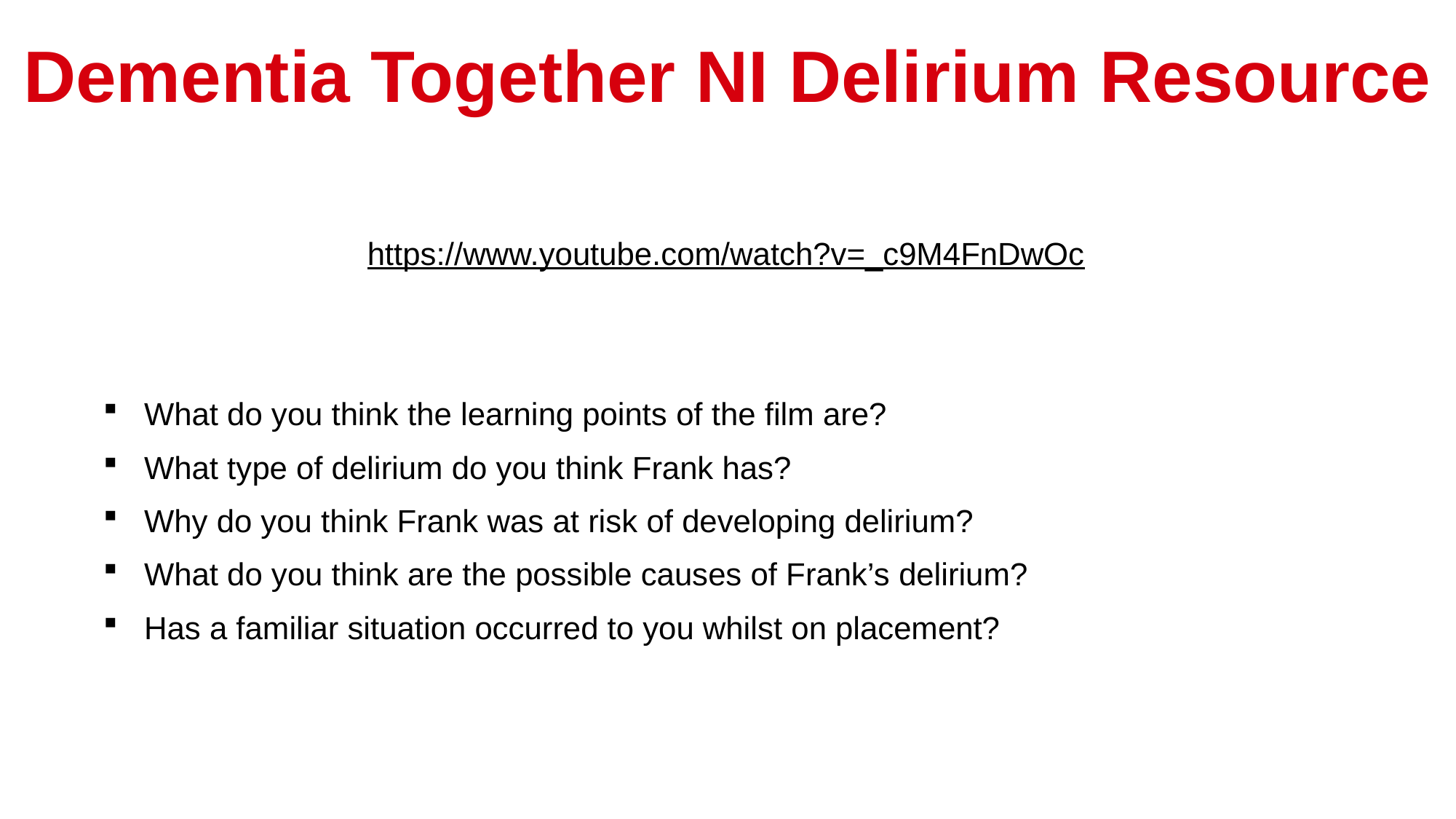

Dementia Together NI Delirium Resource
https://www.youtube.com/watch?v=_c9M4FnDwOc
What do you think the learning points of the film are?
What type of delirium do you think Frank has?
Why do you think Frank was at risk of developing delirium?
What do you think are the possible causes of Frank’s delirium?
Has a familiar situation occurred to you whilst on placement?

## Slide 17
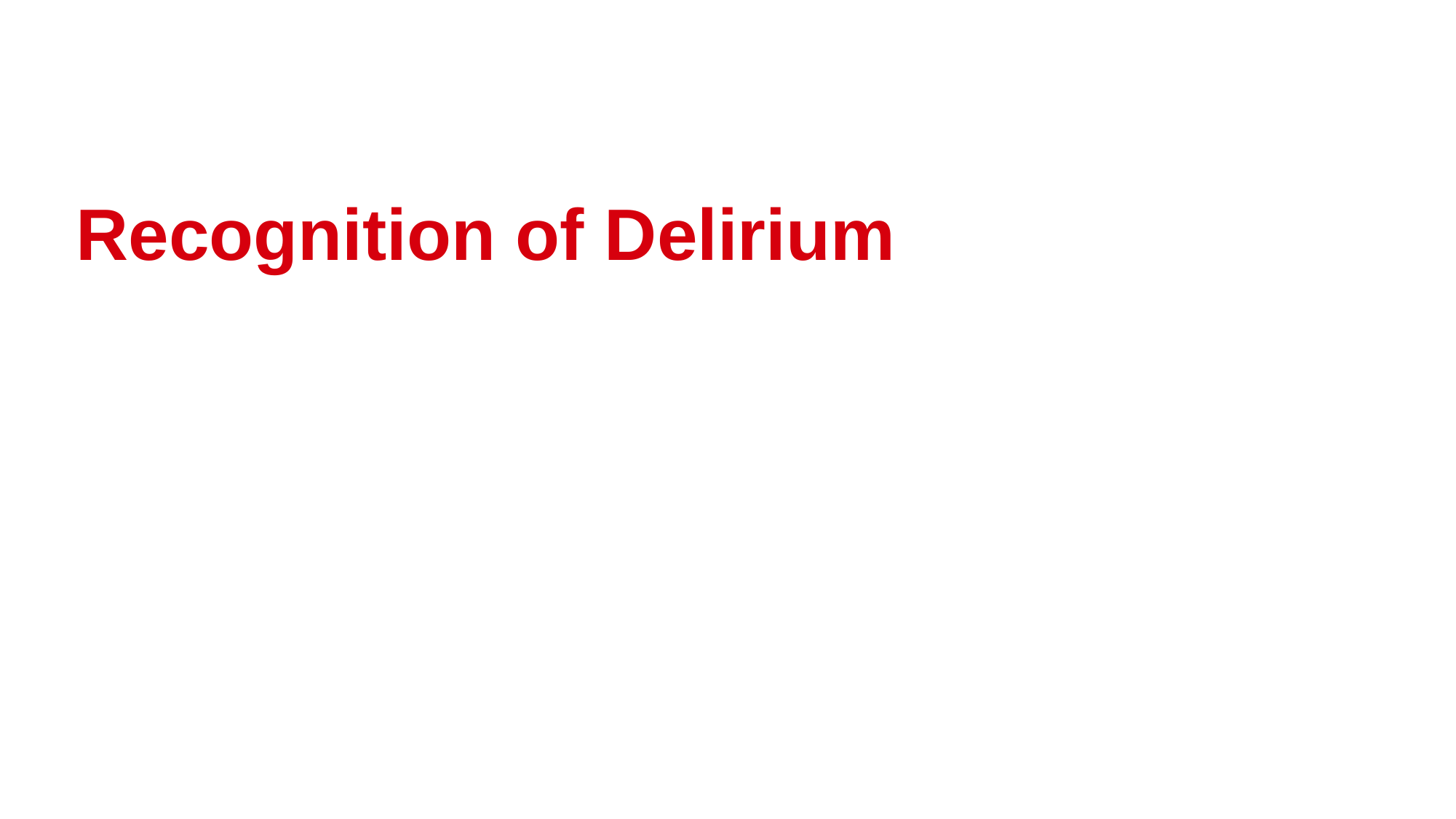

Recognition of Delirium

## Slide 18
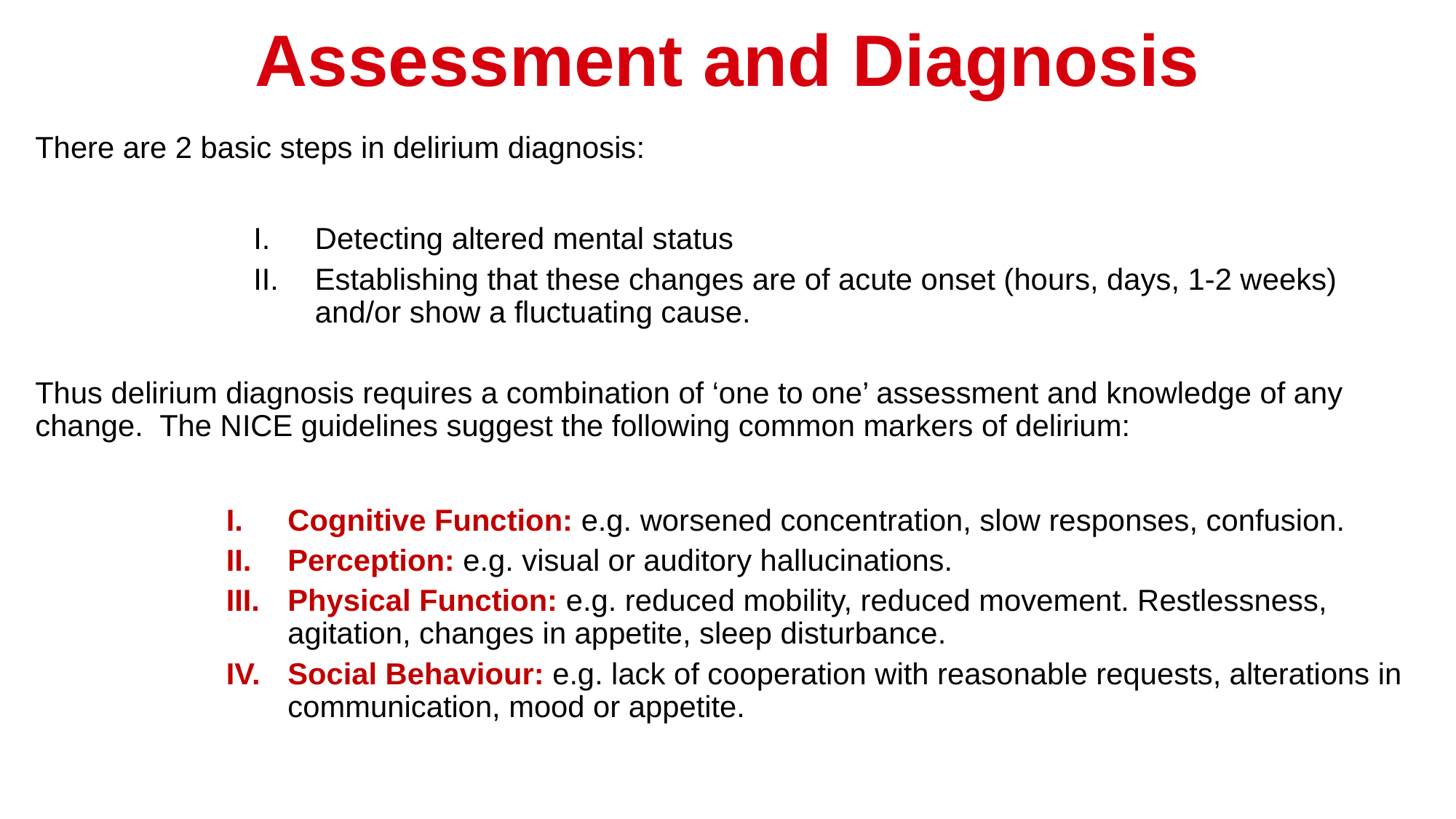

Assessment and Diagnosis
There are 2 basic steps in delirium diagnosis:
Detecting altered mental status
Establishing that these changes are of acute onset (hours, days, 1-2 weeks) and/or show a fluctuating cause.
Thus delirium diagnosis requires a combination of ‘one to one’ assessment and knowledge of any change. The NICE guidelines suggest the following common markers of delirium:
Cognitive Function: e.g. worsened concentration, slow responses, confusion.
Perception: e.g. visual or auditory hallucinations.
Physical Function: e.g. reduced mobility, reduced movement. Restlessness, agitation, changes in appetite, sleep disturbance.
Social Behaviour: e.g. lack of cooperation with reasonable requests, alterations in communication, mood or appetite.

## Slide 19
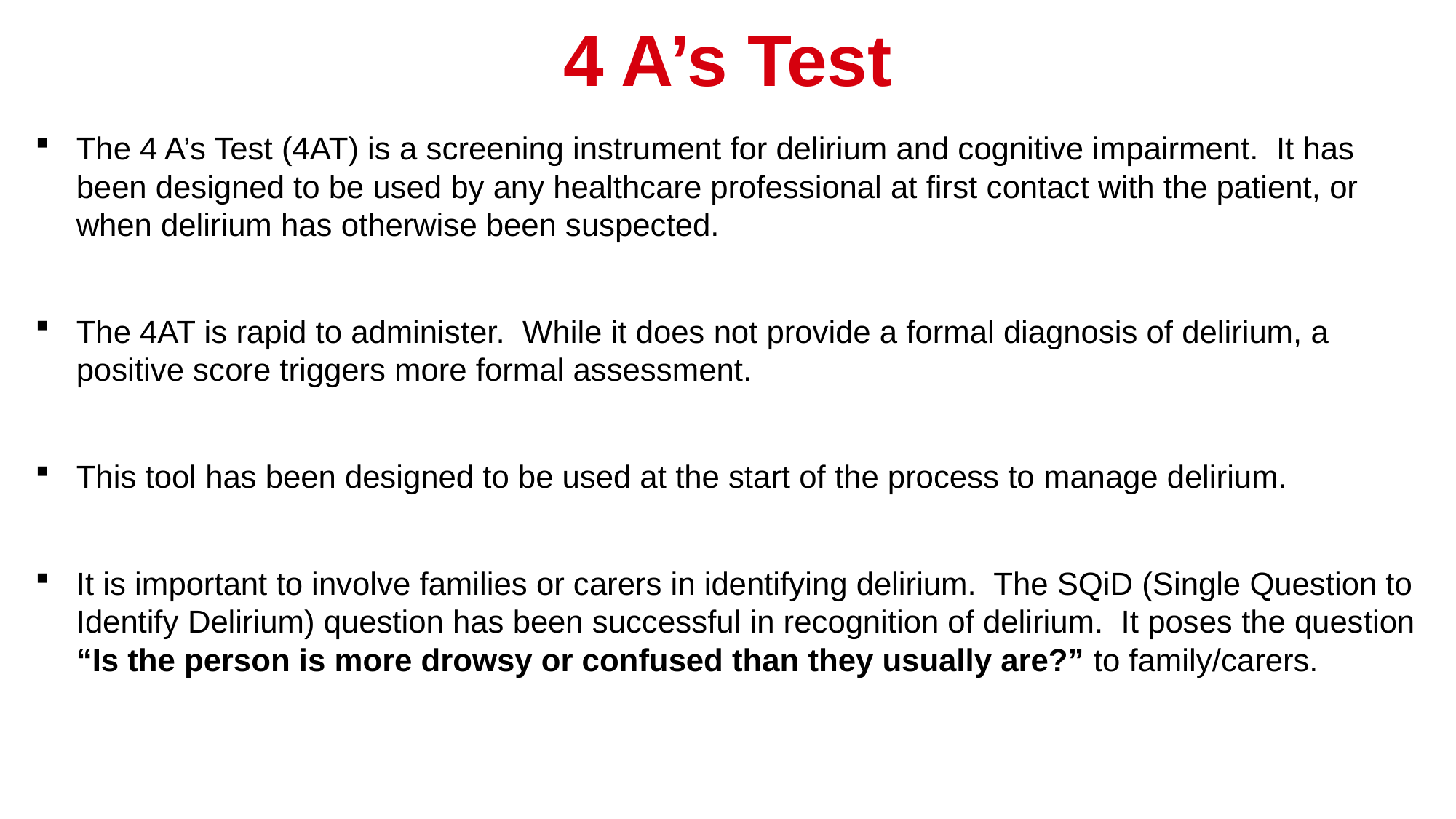

4 A’s Test
The 4 A’s Test (4AT) is a screening instrument for delirium and cognitive impairment. It has been designed to be used by any healthcare professional at first contact with the patient, or when delirium has otherwise been suspected.
The 4AT is rapid to administer. While it does not provide a formal diagnosis of delirium, a positive score triggers more formal assessment.
This tool has been designed to be used at the start of the process to manage delirium.
It is important to involve families or carers in identifying delirium. The SQiD (Single Question to Identify Delirium) question has been successful in recognition of delirium. It poses the question “Is the person is more drowsy or confused than they usually are?” to family/carers.

## Slide 20
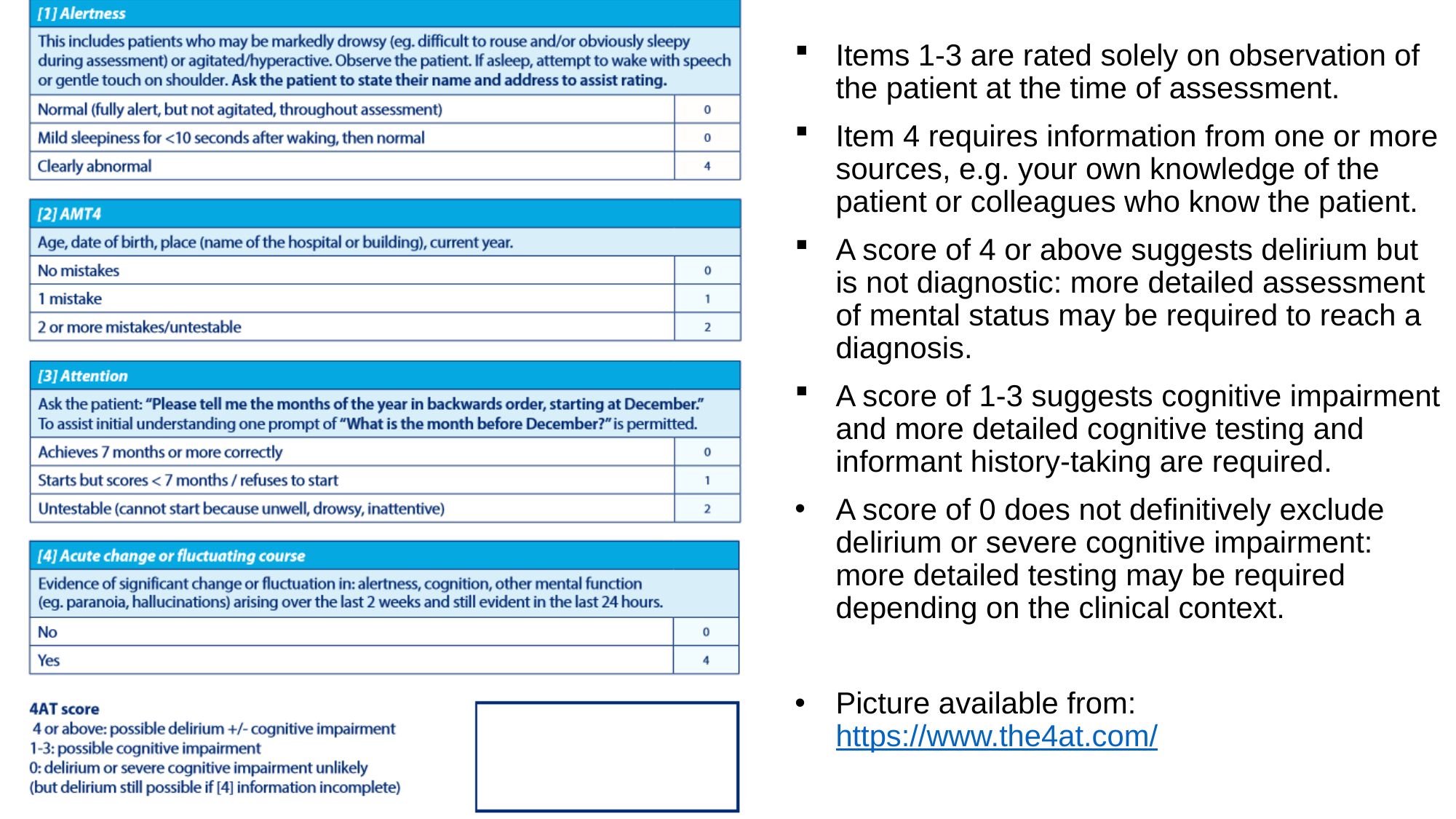

Items 1-3 are rated solely on observation of the patient at the time of assessment.
Item 4 requires information from one or more sources, e.g. your own knowledge of the patient or colleagues who know the patient.
A score of 4 or above suggests delirium but is not diagnostic: more detailed assessment of mental status may be required to reach a diagnosis.
A score of 1-3 suggests cognitive impairment and more detailed cognitive testing and informant history-taking are required.
A score of 0 does not definitively exclude delirium or severe cognitive impairment: more detailed testing may be required depending on the clinical context.
Picture available from: https://www.the4at.com/

## Slide 21
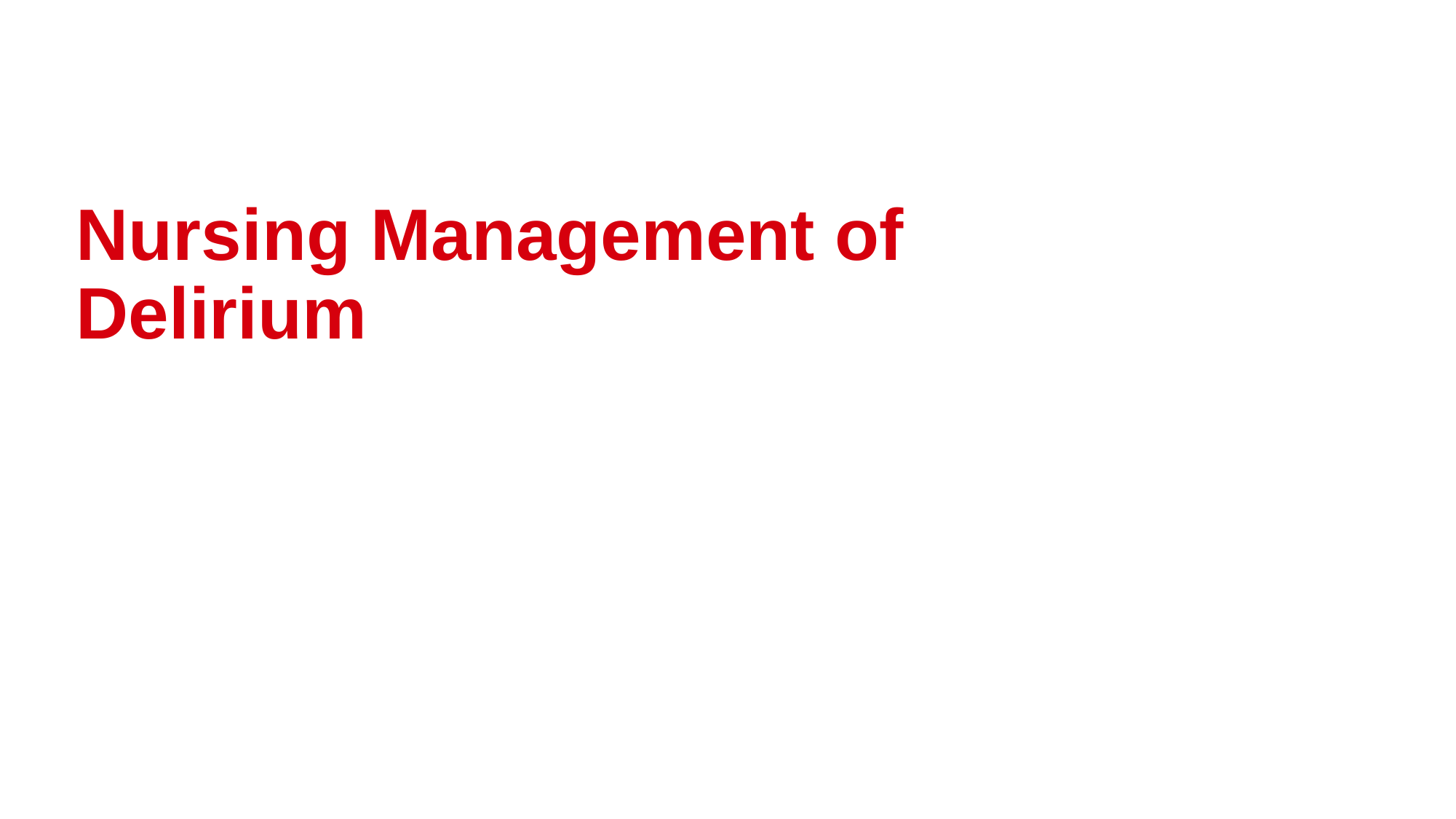

Nursing Management of Delirium

## Slide 22
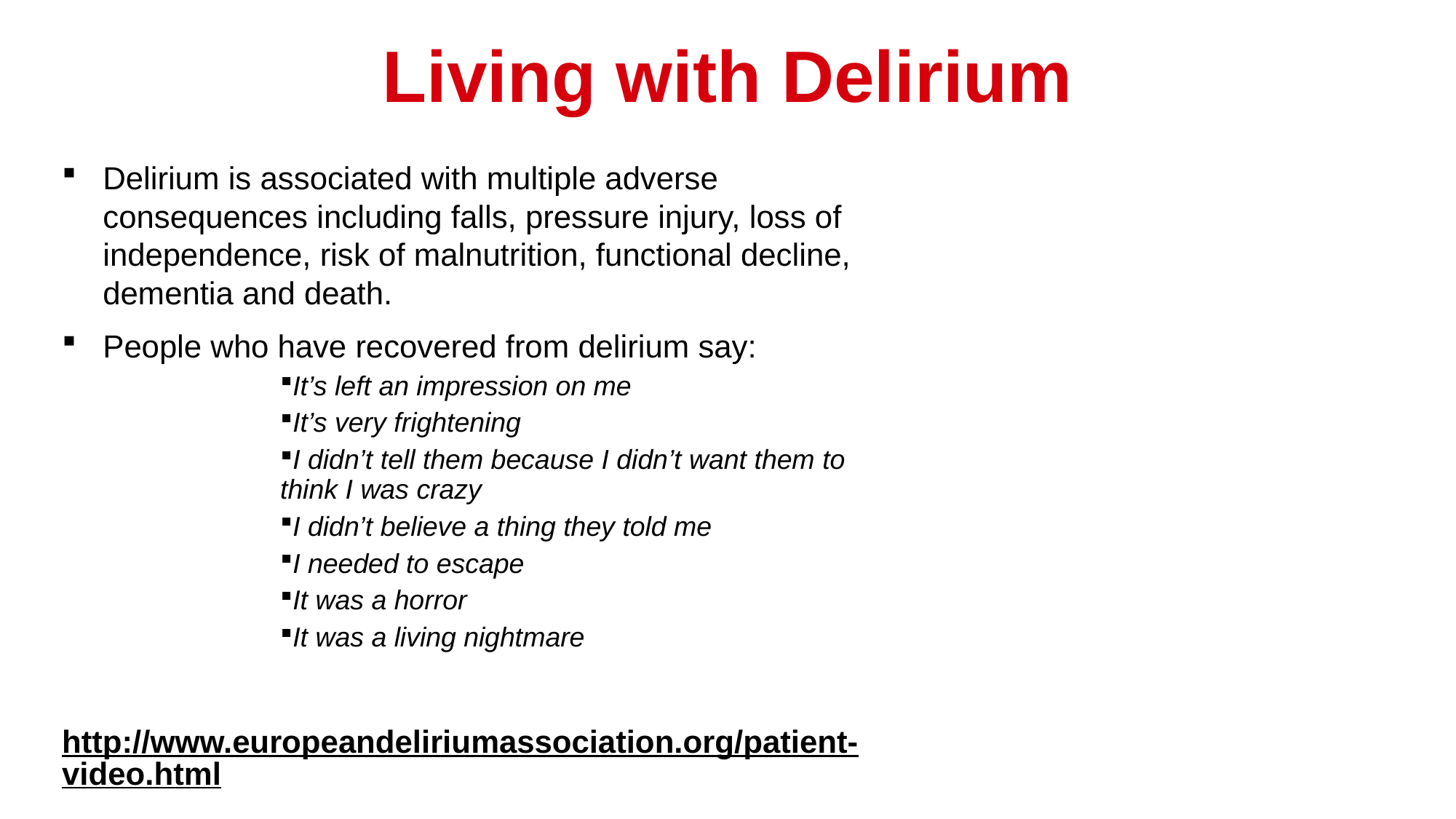

Living with Delirium
Delirium is associated with multiple adverse consequences including falls, pressure injury, loss of independence, risk of malnutrition, functional decline, dementia and death.
People who have recovered from delirium say:
It’s left an impression on me
It’s very frightening
I didn’t tell them because I didn’t want them to think I was crazy
I didn’t believe a thing they told me
I needed to escape
It was a horror
It was a living nightmare
http://www.europeandeliriumassociation.org/patient-video.html

## Slide 23
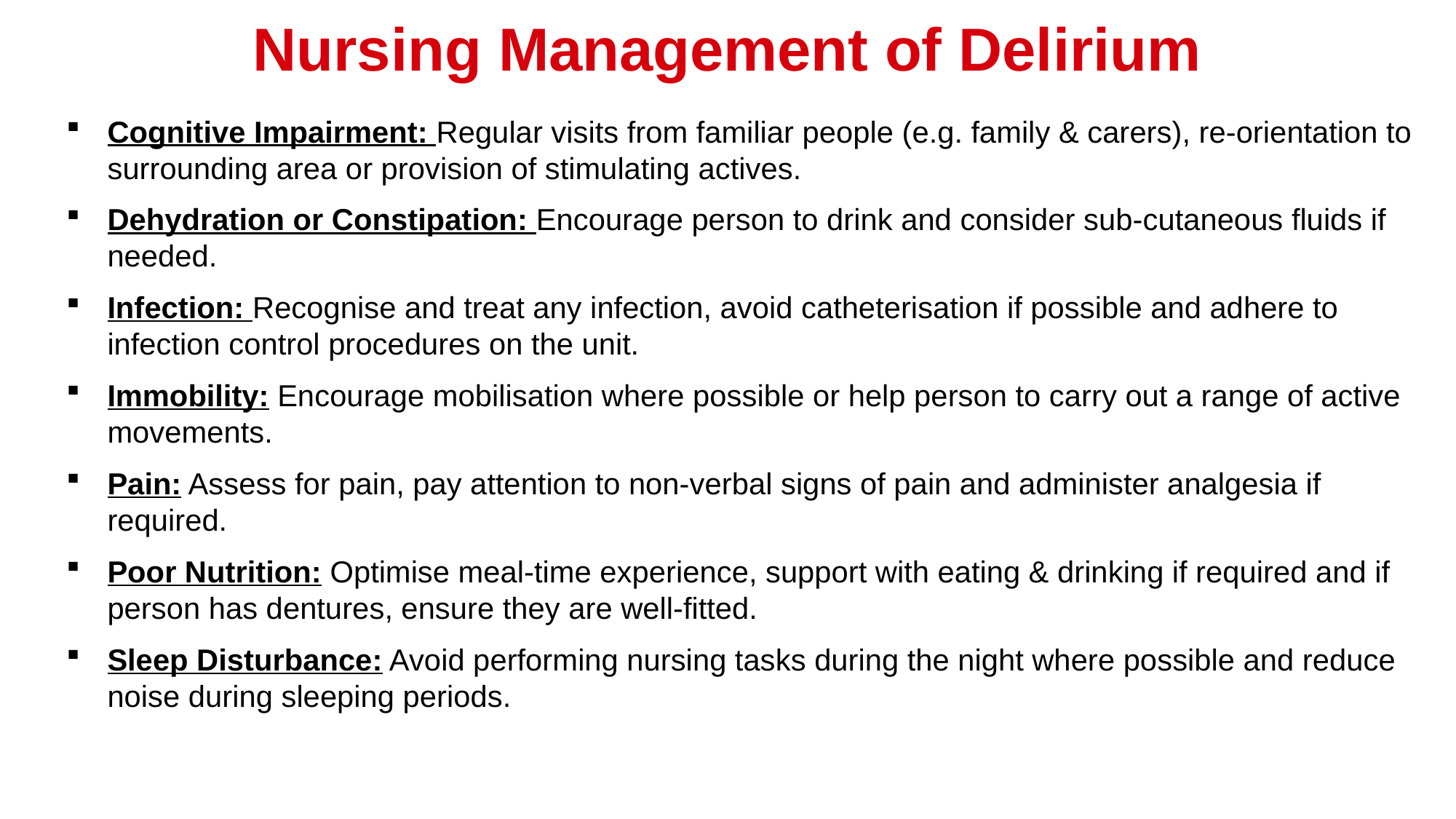

Nursing Management of Delirium
Cognitive Impairment: Regular visits from familiar people (e.g. family & carers), re-orientation to surrounding area or provision of stimulating actives.
Dehydration or Constipation: Encourage person to drink and consider sub-cutaneous fluids if needed.
Infection: Recognise and treat any infection, avoid catheterisation if possible and adhere to infection control procedures on the unit.
Immobility: Encourage mobilisation where possible or help person to carry out a range of active movements.
Pain: Assess for pain, pay attention to non-verbal signs of pain and administer analgesia if required.
Poor Nutrition: Optimise meal-time experience, support with eating & drinking if required and if person has dentures, ensure they are well-fitted.
Sleep Disturbance: Avoid performing nursing tasks during the night where possible and reduce noise during sleeping periods.

## Slide 24
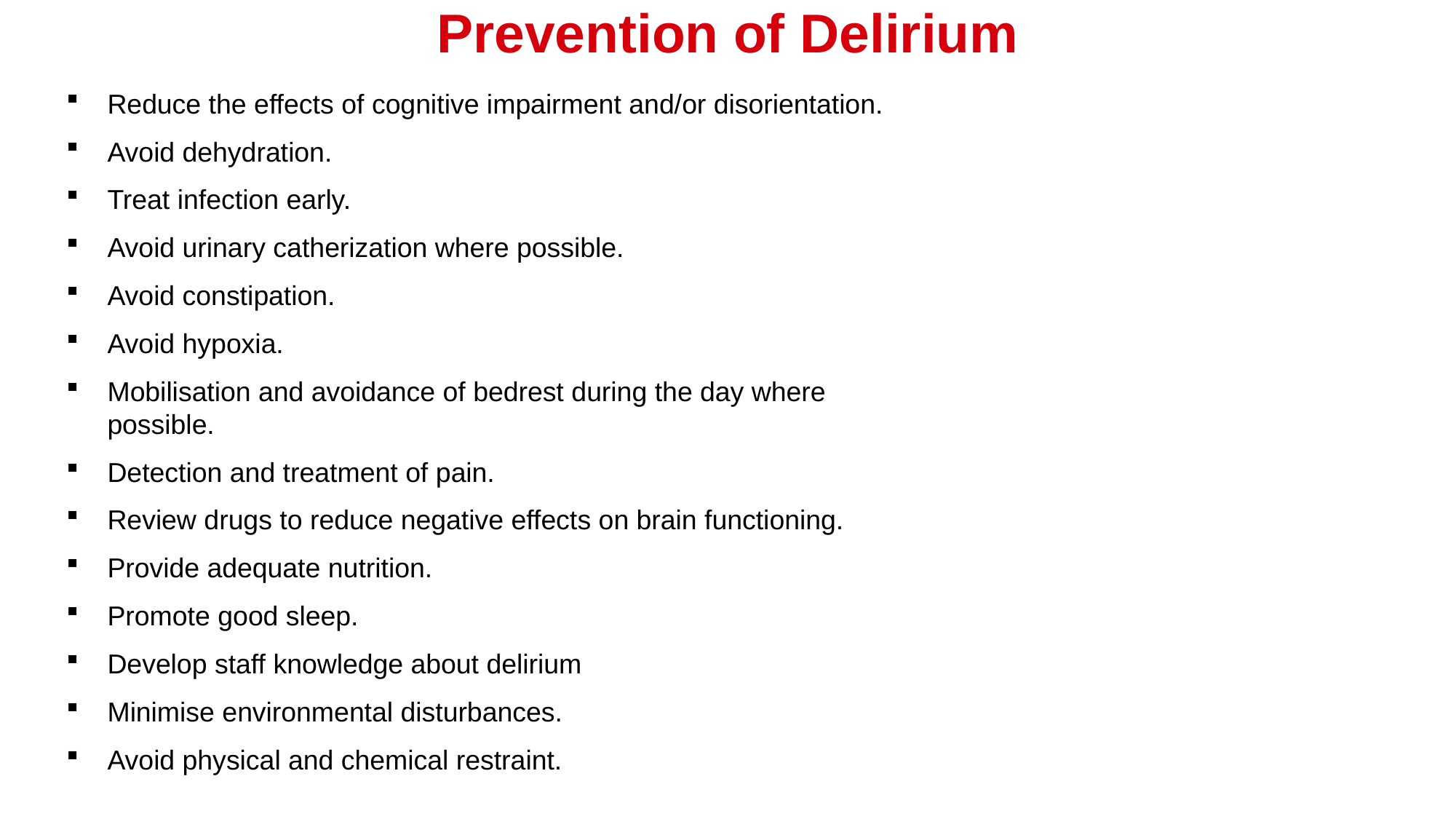

Prevention of Delirium
Reduce the effects of cognitive impairment and/or disorientation.
Avoid dehydration.
Treat infection early.
Avoid urinary catherization where possible.
Avoid constipation.
Avoid hypoxia.
Mobilisation and avoidance of bedrest during the day where possible.
Detection and treatment of pain.
Review drugs to reduce negative effects on brain functioning.
Provide adequate nutrition.
Promote good sleep.
Develop staff knowledge about delirium
Minimise environmental disturbances.
Avoid physical and chemical restraint.

## Slide 25
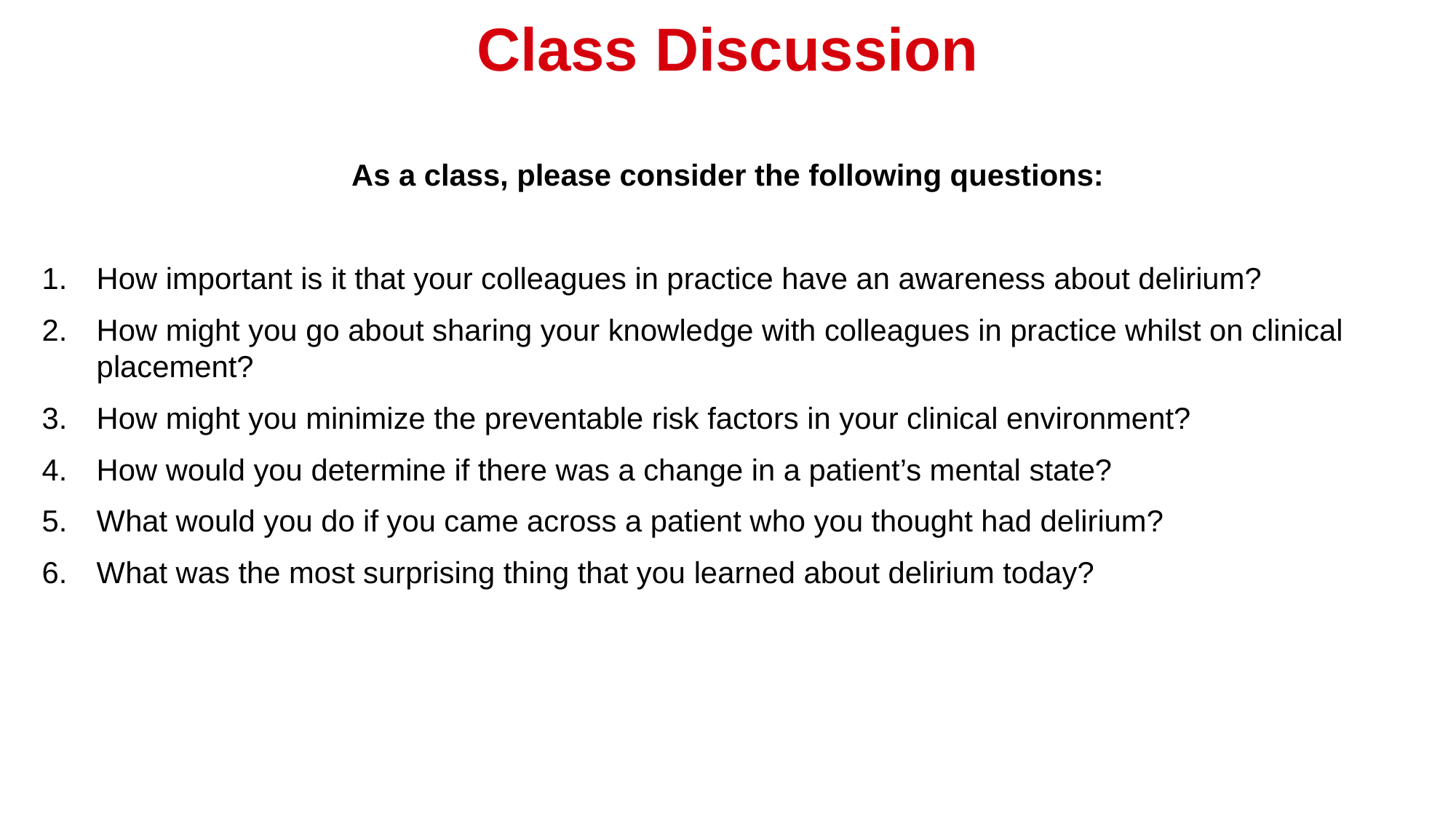

Class Discussion
As a class, please consider the following questions:
How important is it that your colleagues in practice have an awareness about delirium?
How might you go about sharing your knowledge with colleagues in practice whilst on clinical placement?
How might you minimize the preventable risk factors in your clinical environment?
How would you determine if there was a change in a patient’s mental state?
What would you do if you came across a patient who you thought had delirium?
What was the most surprising thing that you learned about delirium today?

## Slide 26
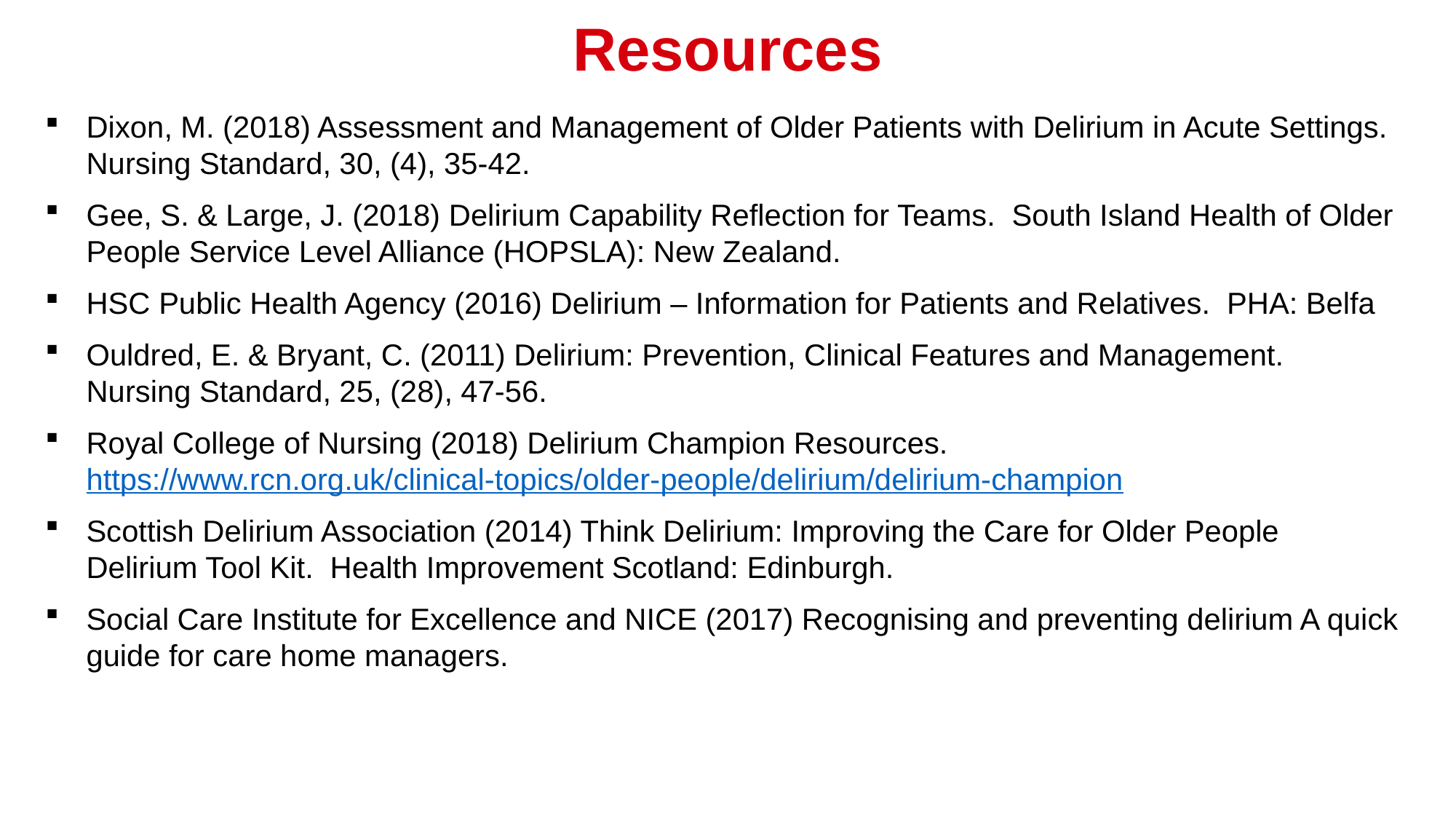

Resources
Dixon, M. (2018) Assessment and Management of Older Patients with Delirium in Acute Settings. Nursing Standard, 30, (4), 35-42.
Gee, S. & Large, J. (2018) Delirium Capability Reflection for Teams. South Island Health of Older People Service Level Alliance (HOPSLA): New Zealand.
HSC Public Health Agency (2016) Delirium – Information for Patients and Relatives. PHA: Belfa
Ouldred, E. & Bryant, C. (2011) Delirium: Prevention, Clinical Features and Management. Nursing Standard, 25, (28), 47-56.
Royal College of Nursing (2018) Delirium Champion Resources. https://www.rcn.org.uk/clinical-topics/older-people/delirium/delirium-champion
Scottish Delirium Association (2014) Think Delirium: Improving the Care for Older People Delirium Tool Kit. Health Improvement Scotland: Edinburgh.
Social Care Institute for Excellence and NICE (2017) Recognising and preventing delirium A quick guide for care home managers.
